# The conceptual framework for a combined food literacy and physical activity intervention to optimize metabolic health among women of reproductive age in urban Uganda

**DOI:** 10.1186/s12889-022-12740-w

**Published:** 2022-02-18

**Authors:** Peter Yiga, Wendy Van Lippevelde, Jan Seghers, Patrick Ogwok, Henry Tafiire, Susan Nakaayi Muluuta, Christophe Matthys

**Affiliations:** 1 Department of Food Technology, Kyambogo University, Kampala, Uganda; 2 Clinical and Experimental Endocrinology, Department of Chronic Diseases and Metabolism, KU Leuven, Leuven, Belgium; 3 Department of Marketing, Innovation and Organization, Ghent University, 9000 Ghent, Belgium; 4 Department of Movement Sciences, KU Leuven, Leuven, Belgium; 5 Department of Endocrinology, University Hospitals Leuven, Leuven, Belgium

**Keywords:** Food literacy, Physical activity, Metabolic health, Women of reproductive age, Intervention mapping protocol

## Abstract

**Background:**

Metabolic health of urban Ugandans, mostly women, has increasingly become sub-optimal. As women are strategic for family behavioral change and do not meet WHO recommendations regarding dietary and physical activity (PA), there is an urgent need for science-based interventions to tackle unhealthy dietary and PA behaviors.

**Objective:**

To develop a food literacy and PA promotion intervention to optimise metabolic health among women of reproductive age in urban Uganda.

**Methodology:**

Steps 1- 6 of the Intervention Mapping protocol were used to design the intervention.

**Results:**

Notable determinants from Step 1 were health/beauty paradox, nonfactual nutrition information, socio-cultural misconceptions around moderate PA, fruits, and vegetables. Others included gaps in food/PA knowledge, skills, and self-efficacy. We hypothesised that changing the overall existing behaviours in one intervention may meet strong resistance. Thus, we decided to go for gradual stepwise changes. Hence in step 2, three behavioural intervention objectives were formulated; (1) women evaluate the accuracy of nutrition and PA information., (2) engage in moderate intensity PA for at least 150 min a week, and (3) consume at least one portion of vegetables and one portion of fruit every day. Based on the food literacy model, intervention objectives were formulated into performance objectives and matrices of change objectives. In step 3 a combination of eleven behavioural change techniques were selected and translated into practical strategies to effect changes in determinants. In step 4, intervention components and materials were developed. The intervention consists of five interactive group sessions, 150 min each. Infographics on benefits/recommendations, vegetable recipes, and practical tips to eat more fruits, vegetables, and to engage more in PA are included. Personalised goals and action plans tailored to personal metabolic health and lifestyle needs, and environmental opportunities form the basis of the intervention. A randomized controlled trial is being conducted to evaluate the intervention (https://clinicaltrials.gov/ct2/show/NCT04635332).

**Conclusions:**

The intervention is novel, based on a holistic food literacy model. The intervention is built on determinants specific to urban Uganda, evidence based behavioural change theoretical models and techniques, detailing the hypothesised behavioural change mechanism. If effective, an evidence-based intervention will become available for reference in urban Uganda.

**Supplementary Information:**

The online version contains supplementary material available at 10.1186/s12889-022-12740-w.

## Background

Over the last two decades, metabolic health of Ugandans, particularly residing in urban areas, has taken an undesirable trajectory [1, 2]. Sub-optimal metabolic health is a major risk factor for non-communicable diseases (NCD). NCDs currently account for almost 50% of all deaths and disability in Low and Middle-Income Countries (LMIC) [1, 3–5]. Sub-optimal metabolic health is mainly related to overweight and obesity [6]. Prevalence of overweight in urban Uganda is higher among women than men at 28.8% and 15.8%, respectively [2]. Prevalence of obesity is respectively 15.6% in women and 3.1% in men [2]. Evidence links maternal obesity to lifelong negative health outcomes not only for the mother but also the offspring [7]. Maternal obesity before pregnancy predisposes the mother to hypertension, pre-eclampsia and gestational diabetes which affect foetal energy metabolism [7]. In-utero exposure to obesity results in epigenetic processes, such as alterations in DNA methylation and alterations in gut microbiome [8, 9]. The increasing maternal overweight and obesity prevalence in urban Uganda translates in an intergenerational obesity and NCD burden. The rise in prevalence in obesity and overweight is projected in a prevalence of raised fasting glucose (4.8%) and total cholesterol (11.5%) among urban dwellers compared to (3.1%) and (5.6%) respectively in rural dwellers [2]. The increasing burden of NCD and related metabolic clinical indicators in urban Uganda reflect an urgent need to develop efficient preventative strategies [6, 10].

Key modifiable risk factors for overweight and obesity are unhealthy dietary patterns and low levels of PA [11, 12]. Dietary patterns of urban Ugandans show poor alignment with World Health Organization (WHO) recommendations [2, 13]. It is estimated that 90% of urban Ugandans do not consume the recommended 400 g of fruits and vegetables daily. Studies show that PA levels are decreasing in urban Uganda, especially among women [2, 14]. Clinically relevant improvements in metabolic health can be achieved by routine, moderate- intensity PA and/or dietary interventions consistent with WHO health recommendations [13, 15]. However, in urban Uganda and sub-Sahara African (SSA) settings, there is hardly any evidence of behavioral change strategies which could be applied to align prevailing dietary and PA behaviors to WHO recommendations [16]. A “copy/paste” approach of the Western world interventions is inappropriate as different determinants play a role [16, 17]. Hence, there is a need for theory and evidence-based lifestyle interventions designed in consideration of contextual determinants of urban SSA.

Interventions targeting women are likely to effect changes across the entire family in SSA. Women of Reproductive Age (WRA), are not only the most vulnerable health wise [7] but are also the most strategic for family behavioral change in the SSA as they are the gatekeepers of the home food environment. In SSA, household lifestyle decisions are made by mostly women, and they directly influence the family’s dietary and PA behaviours [16–18]. Hence, we have designed a behavioural change intervention to optimise dietary and PA behaviors and subsequent sub optimal metabolic health among WRA in urban Uganda. It is recommended that health promotion interventions are systematically designed based on theory and empirical evidence and reported to ensure clarity about the used behaviour change techniques (i.e. the active intervention ingredients) [19, 20]. However, across majority of the existing interventions, it is often unclear how and where theory and empirical evidence are applied [19, 21]. The purpose of this paper is to describe the systematic application of theory and evidence to develop an intervention to optimise dietary and PA behaviors and metabolic health among WRA in urban Uganda, using the Intervention Mapping protocol (IM) [20].

## Methods

The intervention was designed following the IM protocol [20]. The IM protocol consists of a systematic six-step, iterative method to intervention development and evaluation [20]. The steps were accomplished by a strategic planning group, divided into two small groups to enable time and cost-saving working while ensuring quality output. Planning group I consisted of; professors with expertise in behavioral nutrition and PA (n=4), a PhD researcher and MSc. Human Nutrition students (n=4). Planning group II consisted of female representatives from the collaborating religious’ institutions within the target community in urban Uganda (n=4). Planning group I worked behind the scenes to accomplish the different tasks and presented drafts to planning group II for feedback on community fitness. The intervention was developed during the period April 2018 to May 2019 (step 1) and June 2019 to October 2020 (step 2 to 5).

### Step I: Needs Assessment

In step I; a systematic review [16] and a qualitative study [17] were conducted to synthesis determinants of dietary and PA behavior among WRA in urban SSA and Uganda respectively. Step I also encompassed assessment of community assets. The environmental asset assessment framework for a health promotion program planning by Springer and Evans [22] was used. The assessment encompassed brainstorming workshops with the planning group I, meetings with strategic community leaders and focus group discussions (FGDs) with the target group (urban Ugandan women aged between 18 and 45 years). The FGDs were designed as part of the qualitative study [17] (questions on implementation and design were included – Additional file 1). Questions on the implementation and design were not part of the previously published qualitative study [17] and are reported in the current paper. Consolidated criteria for reporting qualitative research is followed as a guideline to report these FGDs results [23]. The methodology is reported in Yiga et al., [17]. Details of the different methodological aspects of the sub studies conducted during intervention development are elaborated in Additional file 2.

### Step II: Formulation of behavioral intervention, performance, and change objectives

Behavioral intervention, performance, and change objectives were formulated based on the results of step I. Performance objectives (POs) are the sub behaviors that must be accomplished by the target group to achieve the behavioral intervention objectives. Then, the determinants considered to have a strong influence on accomplishing the specific POs were identified and selected. Selection of the determinants was based on the relevance (strength of relationship with the behavior) and changeability (extent to which a determinant can be changed). Identification and selection of the determinants were guided by the results of step I. POs were linked to the determinants, to formulate matrices of the change objectives. The change objectives specify what needs to be changed in determinants to achieve specific POs. For each behavioral intervention objective, a matrix of the change objectives was built.

### Step III: Selection of theory - based methods and practical strategies

The change objectives were organized per determinant. The behavioural change techniques (BCTs) were then matched to the determinants. Selection of the BCTs was informed by expert opinion (iterative brainstorming workshops with planning group I), scientific evidence and behavioural theories. The taxonomy of evidence-based BCT compiled by Michie et al., [24] and the summary of theoretical methods provided by Eldredge et al., [20] were used. Selected BCTs were then operationalised into the practical intervention strategies required to accomplish the change objectives. Preconditions for applicability of the BCTs were critically considered in the translation of BCTs to practical strategies [20]. To arrive at the practical intervention strategies, a brainstorming workshop was held with planning group II. The workshop was complemented with data from FGDs with the target group (from step I). The output from the latter workshop and FGDs was fine-tuned through iterative brainstorming workshops with planning group I and the literature review of how the selected BCTs are applied in practice. The final output from step III is the hypothesised intervention logical model, built by combining outputs from step I to III.

### Step IV: Development of the intervention programme

Information from step III was compiled into the intervention scope and sequence. Information generated from step I to III was used to design the intervention scripts and documents. Intervention materials were pretested in planning group II and additional members of the target group and improved accordingly. Due to the Covid -19 situation the pretesting was executed through one-on-one consultations through telephone calls and WhatsApp image exchanges. Step IV also encompassed assessment of the community to identify a potential intervention delivery channel. The assessment of community assets in step I was used to identify the delivery channel.

### Step V: Development of an adoption and implementation plan

Step V focused on the planning of the implementation plan of the intervention. The FGDs with the target group and meetings with planning group II provided important information about the factors that would enhance implementation of the intervention. Methodologically following the IM protocol, the earlier steps (I to IV) are repeated to create an intervention for adoption, implementation, and sustainability. However, creating an intervention for adoption and sustainability was beyond the scope of our resources and timeline. Hence, in step V we adapted the IM protocol to focus on the development of the implementation plan to evaluate the developed behavioral change intervention through a proof-of-concept study.

### Step VI: Development of an evaluation plan

A plan to evaluate the effectiveness of the intervention was developed. We followed the CONSORT recommendations for cluster randomized trials to design a randomized control trial [25].

## Results

### Step I: Needs assessment

**Fig. 1:**
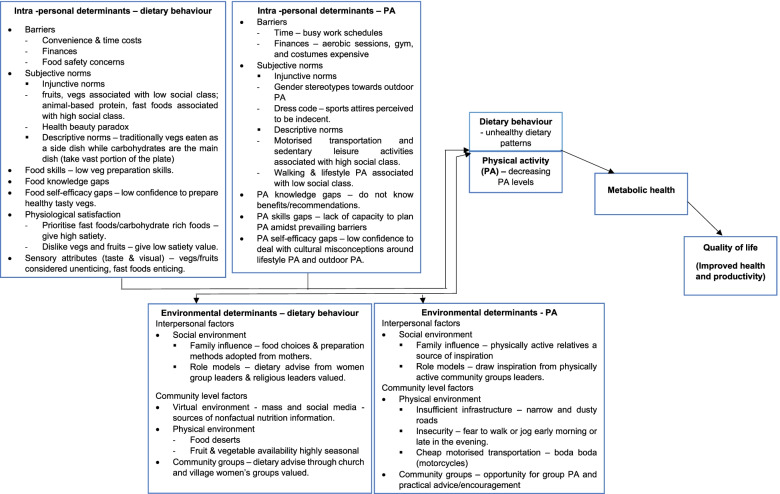
Logical model of needs assessment, summarizing the personal and environmental determinants of dietary and PA behavior in urban Uganda. Adapted from Yiga et al., [16] and Yiga et al., [17]

Findings from our systematic review [16] were used to design a theoretical framework for the qualitative study [17]. Notable determinants identified in the systematic review were financial and time limitations, health/beauty paradox (= overweight/obesity as a sign of beauty and wealth), and lack of knowledge, self-efficacy, and skills. Qualitative study findings re-affirmed the systematic review findings concerning health/beauty paradox, knowledge, self-efficacy, and skills gaps. In addition, the qualitative study showed socio-cultural misconceptions around lifestyle PA, fruits, vegetables, and habitual orientation towards carbohydrate foods. We also found that there is a high trust in nutrition information shared on social and mass media, yet skills to evaluate this nutrition information are limited. Figure 1 below shows the logical model of needs assessment, summarises the determinants of dietary and PA in urban Uganda [16, 17].

### Step II: Formulation of behavioral intervention, performance, and change objectives

 We hypothesised that changing the overall existing behaviours towards WHO healthy lifestyle guidelines in one intervention may meet strong resistance and thus may not be effective. For example, the planning group hypothesised that due to the existing health/beauty paradox and habitual orientation towards carbohydrate rich foods, interventions focusing directly on weight loss and reduction of portion sizes of foods rich in carbohydrates may meet strong resistance. Therefore, we decided to go for more feasible gradual changes able to enact clinically relevant metabolic improvements. We hypothesised that increased consumption of vegetables and fruits will indirectly translate into reduction of portion sizes of carbohydrate rich foods. In line with WHO health recommendations, the intervention aims to stimulate WRA to consume at least 400 g fruits and vegetables [13]. Moderate intensity PA that can be incorporated in daily life activities may be the achievable type of PA among WRA compared to structural high intensity PA [26]. Non-factual nutrition information influences dietary and PA behaviors in urban Uganda [17]. Thus, we decided to supplement the intervention with a component on information evaluation; to enact ability to distinguish evidence-based information from nonfactual information.

Accordingly, three behavioural intervention objectives were formulated.

1. Women evaluate the accuracy of food, nutrition, and PA information.
2. Women engage in moderate intensity PA for at least 150 min a week.
3. Women consume at least one portion of vegetables and one portion of fruit every day.

Table 1 shows the behavioral intervention objectives, subdivided into POs providing the answer to the question; “what do the participants of the intervention need to do to achieve the behavioural objectives”. The model of food literacy [27] guided the formulation of POs. Food literacy is the interrelated combination of knowledge, skills and self-efficacy to (i) plan, (ii) select, (iii) prepare, (iv) eat food with the ultimate goal of developing a lifelong healthy, sustainable and gastronomic relationship with food within the prevailing environment [27, 28]. The POs were based on the above mentioned four components of food literacy (plan, select, prepare, and eat). For PA, a similar model was adopted, where “eat” was replaced with “do”, that is; plan, select, prepare, and do. The model of food literacy was chosen as it is a holistic behavior change model focusing on a “how to do approach” to initiate and sustain healthy eating habits [27, 28]. Evidence shows a positive association between food literacy and healthy dietary behaviors, particularly increased intake of vegetables and fruits [29, 30]. Table 2 shows the determinants considered to have a strong influence on accomplishing the created POs. Matrices of change objectives are presented in Additional file 3.

**Table 1:** Behavioural intervention objectives subdivided into performance objectives

Women evaluate the accuracy of food, nutrition, and PA information
PO1: Women search for food, nutrition, and PA information. PO2: Women judge the accuracy/correctness of food, nutrition, and PA information
Women engage in moderate intensity PA for at least 150 min a week
PO1: Women plan specific moderate intensity PA moment in their daily schedule
PO2: Women execute the planned specific moderate intensity PA moment in their daily schedule
PO3: Women maintain the newly incorporated moderate intensity PA moment in their daily schedule
Women consume at least one portion of vegetables and one portion of fruit every day
PO1: Women decide/plan to eat more fruits and vegetables
PO2: Women buy fruits and vegetables
PO3: Women prepare family meals containing vegetables and fruits
PO4: Women eat vegetables and fruits in varying environments (traveling, at work)
PO5: Women maintain newly learned buying, preparation and eating habits

**Table 2:** Determinants of performance objectives for behavior intervention objectives

Determinant	Relevance	Changeability	Evidence for importance
Determinants of performance objectives for behavior intervention outcome 1			
Knowledge	+++	+++	Knowledge, skills and self-efficacy important in developing information evaluation behavior (Vidgen HA [27]; Perry EA [28]). Gaps from needs assessment [16, 17]
Skills	+++	++	
Self-efficacy	+++	++	
Determinants of performance objectives for behavior intervention outcome 2			
Knowledge	+++	+++	Knowledge gaps (needs assessment) [16, 17]. Precondition for personal attitude and intention development (theory of planned behavior) [31]
Skills	+++	++	Skills gaps (needs assessment) [16, 17]. Skills precondition for self-efficacy and attitude [31]
Self-efficacy	+++	++	Low self-efficacy (needs assessment) [16, 17]. Self-efficacy is determinant for the precursors of behavior – intention, preparation to act, but it can also directly influence implementation & maintenance of behavior (addresses both perceptions & reality). Important in PA [26]
Subjective norms	+++	+ +	Social misconceptions (needs assessment) [16, 17]
Social support	+++	++	Social misconceptions (needs assessment) [16, 17]
Barriers (busy work schedules and finances)	+++	+	Social misconceptions (needs assessment) [16, 17] – we do not work on this determinant directly in our intervention. We rather equip participants with practical plans to have PA within existing schedules and finances
Physical environment	+++	+	Social misconceptions (needs assessment) [16, 17] – we do not work on this determinant in our intervention. We lack resources to improve it.
Determinants of performance objectives for behavior intervention outcome 3			
Knowledge	+++	+++	Knowledge gaps (needs assessment) [16, 17]. knowledge important in dietary decision making [27, 28, 32]
Skills	+++	++	Skills gaps (needs assessment). Skills precondition for self-efficacy. Important in dietary decision making [27, 28, 32]
Self-efficacy	+++	++	Low self-efficacy (needs assessment) ^[[[[[[ [25, 28]]]]]]]^. Self-efficacy is determinant for the precursors of behavior – intention, preparation to act, but it can also directly influence implementation & maintenance of behavior (addresses both perceptions & reality) ^[[[[[[ [31]]]]]]]^. Important for dietary behavior [27, 28, 32]
Subjective norm	+++	++	Social misconceptions (needs assessment) [16, 17]
Social support	+++	++	Social misconceptions (needs assessment) [16, 17]
Barriers (time constraints, finances & food safety concerns)	+++	+	Social misconceptions (needs assessment) [16, 17] – we do not work on this determinant directly in our intervention. We rather equip participants with practical tips to increase fruit and vegetable intake within existing schedules and finances
Physical environment	+++	+	Social misconceptions (needs assessment) [16, 17] – we do not work on this determinant in our intervention. We lack resources to improve it. We rather equip participants with practical tips to increase fruit and vegetable intake within prevailing environment.

### Step III: Selection of theory-based methods and practical strategies

We aimed to create an intervention capable of initiating and sustaining behaviour change. Eleven BCTs scientifically shown to enact changes in knowledge, skills, self-efficacy, subjective norms, and social support were selected, Additional file 4. The selected BCTs are supported by the self-regulation theory and self-determination theory which specifies the need for autonomy, competence, and relatedness to attain a positive behaviour change [33, 34]. Accordingly, our intervention aims to create behavioural change through enacting autonomy, competence, and relatedness. Providing information coupled with motivation interviewing creates a positive intention [35]. Implementation intentions can be achieved through goal setting [24, 34, 35]. Goal setting necessitates competence, which we hypothesised to be attained through a combination of (i) action planning; (ii) guided practice; ii) self-monitoring; iv) feedback on performance and v) planning of coping plans [24, 26, 34–36]. To sustain the behavioural goals requires relatedness, which can be achieved using a combination of social support, role modelling, feedback, planning coping responses and motivation interviewing [20, 24, 34].

The selected BCTs were then operationalised into practical strategies. BCTs; motivational interviewing, role modelling, feedback, guided practice, social support through exchanging ideas and planning coping responses were translated into interactive group-based sessions. Brainstorming workshops with planning group II and FGDs with target group revealed that group sessions may be the best strategy to deliver the intervention in this setting.

“Through education sessions, like you come in this group and give us a health talk, like the way you have come, you teach us and then us we can go and teach our other friends out there. Like for us every Tuesday we be meeting here, very many of us, so if you say you will give us one Tuesday in a week or month, or the last Tuesday of a month and you come and teach us”. “It would be very nice, because literally I share the information with others, so it will move, it moves much faster, because these groups are not only here, but also have these groups in other dioceses, so we can go visit them, and the teach them, but in health centers you only visit when you’re sick”. “Yes it helps, what I know is good, I wish it for my friends and we act as a support for each, and we as well spread it to other groups, example of myself, I used to never eat pumpkin, but I got it from these ladies, that this pumpkin is good and with time I gradually started to eat it until it become part of my diet”, participants in FGD 4 and 6.

Additionally, a recent systematic review shows that diet and PA interventions delivered through group sessions are effective in promoting clinically relevant weight loss [34]. These groups provide opportunities for social support, experience sharing, and may create a motivating atmosphere [22, 34]. Our needs assessment as well revealed that the community and church small groups are an opportunity to share dietary and PA counselling [16, 17]. Our environmental asset assessment revealed existence of women groups within religious structures. Existing groups boosts social cohesion, a facilitator for behavioural change [22].

The reading culture of Ugandans is low.

“We need more of practical, and also the pamphlet, some of us don’t really understand so much, but if it brings out the picture very well, even I can pick interest in it”. “Pamphlets, some people are lazy to read”, participants in FGD 5.

So, the BCT of “providing information through imagery” was translated into infographics with less text and more locally recognisable visuals. Evidence as well shows that visuals increase attention, interest, and credibility of the messages [20].

During FGDs with the target group, participants emphasised the need for practical vegetable preparation skills.

“like we are trying to reduce cooking oil and other stuff from our daily life, so maybe we meet in a group, there is a demonstration whereby some food stuffs are prepared in the best possible way which is to the taste, and people learn how to prepare them, because most of us, do not know how to cook, that is the truth, but somebody may not even fry food, but it tastes so good, if you know how to mix the ingredients and so on. Yes, include cooking demonstrations”, participants in FGD 2.

**Fig. 2:**
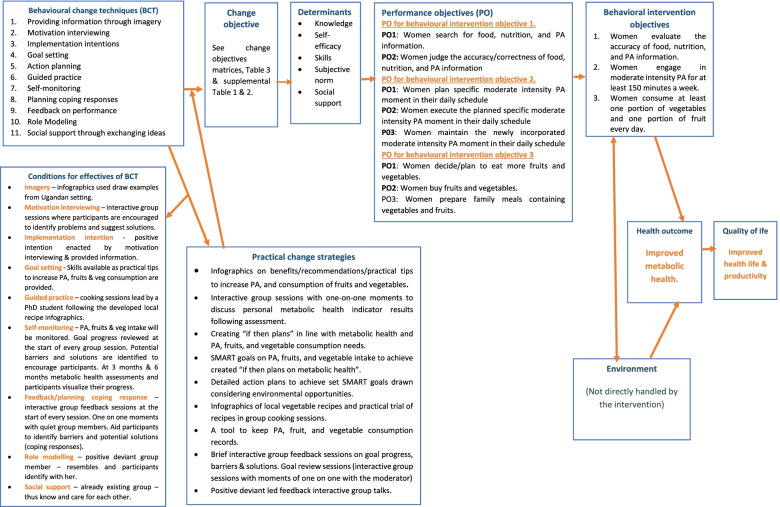
hypothesised intervention logical model for behavioural change (conceptual framework for the intervention)

Hence, BCT of “guided practice” was specifically translated into a practical vegetable group cooking session. We also included vegetable recipes based on locally available vegetables in the intervention infographics. Intervention strategies linked to personal metabolic health and lifestyle needs, and environmental opportunities may help drive behaviour change and positively influence health outcomes [37]. Thus, BCT of; implementation intentions, goal setting and action planning were translated in to; (i) creating “if then plans” in line with metabolic health, (ii) SMART fruit/vegetable/PA goals, detailed action plans to achieve set SMART goals drawn considering environmental opportunities. Figure 2 below shows the hypothesised intervention logical model (conceptual framework) of behavioural change. Practical strategies built from BCT are hypothesized to effect changes in the change objectives, which in turn translate in changes in the determinants. Changes in the determinants in turn result in attainment of POs and corresponding behavioural intervention objectives.

### Step IV: Development of the intervention programme

**Fig. 3:**
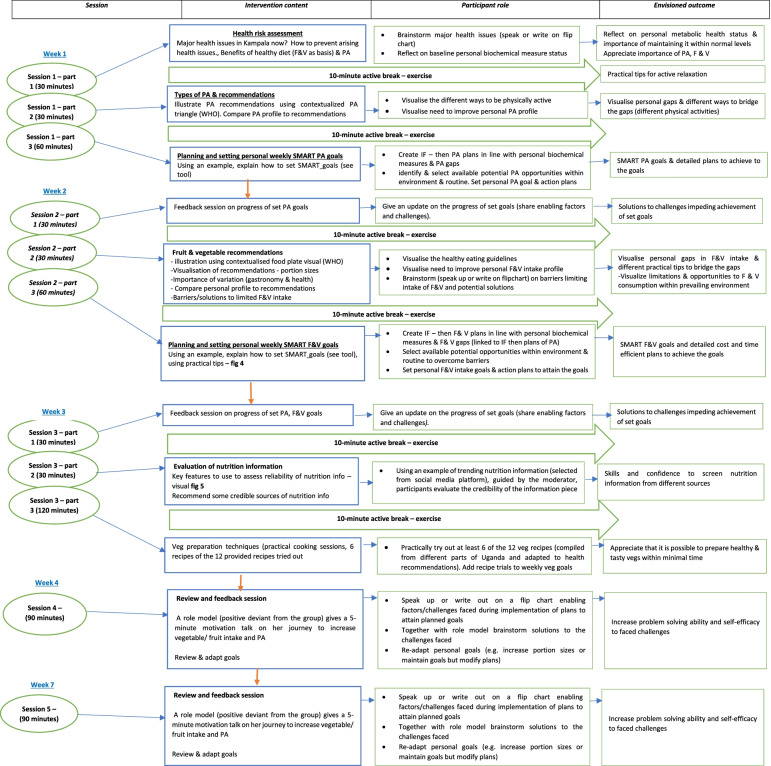
Showing delivery timeline of the intervention sessions, intervention content (organised practical strategies from step III), role of participants, and anticipated outcome per session

The practical strategies were built into the intervention scope and sequence, Additional file 5. The intervention consists of five interactive group sessions, 150 min each, Fig. 3. A booklet (infographics); on benefits/recommendations, local vegetable recipes, and practical tips to eat more fruits, vegetables and do more PA is included as a guide, Additional file 6. Tools to assess PA and food environment for opportunities were included, Additional file 7. As well a self-monitoring tool for PA, fruit and vegetable intake was included for participants to track their behaviour daily goals for use in the feedback sessions, Additional file 8. The infographics were designed with locally recognisable images as cultural relevance of health promotion materials is vital for the success of an intervention [20]. Messages on the infographics were framed in a positive and active tone as evidence shows that positively framed messages are more acceptable [20].

Brain storming workshop with planning group I and FGDs with the target group identified religious institution women group structures as an appropriate potential delivery channel. The women group structures boosts established social networks, community reach (85% Ugandans are Christians) and trust. The channel offers an opportunity for assessing the intervention effectiveness in an unrestricted real-life community setting.

“Religious institutions because they are transparent, religious organizations because they reach out to a bigger community and then they are transparent. The health centers, there is that rudeness, and still for health centers will only meet those people who come to them, but the church, you get a bigger audience”, “Come to churches like this, people really belong to this communities, then you say every third Saturday or Sunday of the month, from 4 to 5 pm, there will always be a nutritional class, for the first-time people may not come, but eventually they come, if it is a free class”, participants in FGD 4.

### STEP V: Adoption and implementation plan

The intervention will be delivered through institutional religious women groups (results of environmental asset assessment framework - see step IV). Through meetings with the strategic community leaders, a collaboration was established with Our Lady of Africa Catholic Parish, Mbuya. Mbuya Catholic Parish has six sub parishes. Within these sub parishes they are existing women groups, and these groups will be utilized for face-to-face intervention group sessions. FGDs with target group and meetings with planning group II pointed at the importance of opinion peer leaders being part of the implementation team.

“Our women group leader has helped us a lot, she taught us the dangers of cooking in polyethene bags and taught us the use of banana leaves, us we had got so much used to using the polyethene bags, she can’t eat the food you have prepared in polyethene bags, even if she visits you and if you have cooked like that, she can’t eat that food. “We have musawo (village health team) in our group, she usually brings for us education sessions on how to eat, she goes a lot for these education sessions and what she learns she brings them back to us”, participants in FGD 6.

Scientific evidence shows that the efficacy and acceptability of health promotion interventions increases if peer opinion leaders within the target group are part of the implementation team [38]. Peer opinion leaders provide entry and legitimacy to the external change agents and may help drive changes in social norms. Selection of peer opinion leaders: the intervention will be delivered within existing women groups. Leaders of these existing groups will be selected to work as peer opinion leaders on the implementation team. The main role and responsibilities peer opinion leaders will be to (i) mobilize fellow women to participate in the intervention, (ii) follow up and (iii) give social support to participating women to attain set intervention goals. Women leaders will be given a two - day refresher training on mobilization and leadership skills, as mobilization is the routine responsibility for women leaders in their usual group meetings. The planning group I designed the sessions to be moderated by health behavior coach (PhD researcher) following the techniques of motivational interviewing [39]. A general guide (scope & sequence) will ensure consistency during the group sessions.

### Step VI: Development of an evaluation plan

#### Study design, setting and timing

**Fig. 4:**
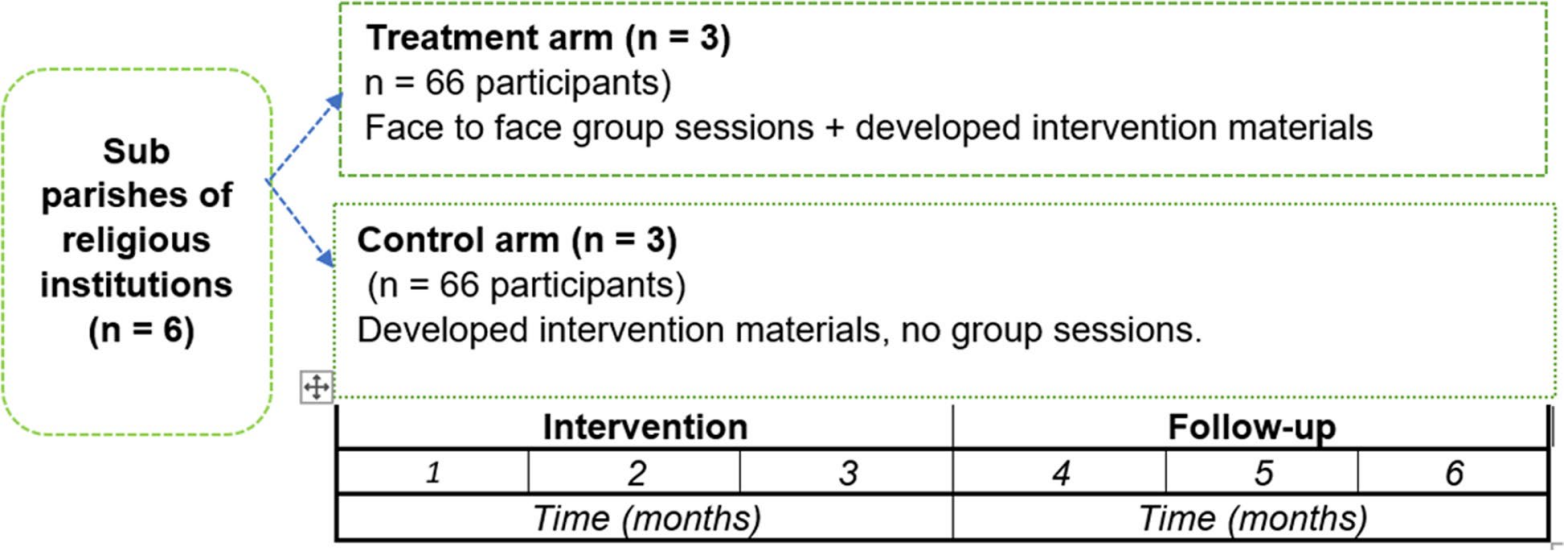
Study design

The effectiveness of the intervention will be evaluated through a cluster-randomized controlled trial. The intervention will be evaluated in Kampala, the capital city of Uganda. The six sub parishes of Mbuya catholic parish will be randomized to treatment and control arms, Fig. 4. The treatment arm will be exposed to both the developed intervention infographics and face to face group sessions while the control arm will only receive the developed intervention infographics. An awareness session will be organized to distribute the infographics to the control arm. Within the sub parishes, there are existing women groups. These existing groups will be utilized for face-to-face intervention group sessions. For the intervention purposes, each group will be limited to a maximum of 14 members. The study period is divided into two phases: a three-month intervention and a three-month post-intervention follow-up phase.

### Recruitment

The PhD researcher and women leaders of existing groups will make presentations about the intervention during one of the routine meetings. Flyers with details of the intervention will be distributed for sharing with members who are absent during the briefing. At the end of the presentations, interested participants will be invited for the first session to test their eligibility to participate in the study. Eligible participants will be provided with an informed consent form to endorse.

#### Inclusion criteria

i) Sex (women),
ii) Age (18 to 45 years),
iii) Central obesity [waist circumference ≥ 80 cm]),
iv) Fluent in either Luganda or English (sessions will be conducted in Luganda/English).
v) Willingness to follow the three-months intervention and three months follow-up,
vi) Willingness to sign the informed consent.

#### Exclusion criteria

i. Being treated for diabetes Mellitus Type 1 or Type 2, hypertension, high cholesterol, or any other cardio-metabolic related disease.
ii. Pregnancy.

### Outcomes

Primary outcome is reduction in waist circumference. Decreases in waist circumference are recommended as critically important treatment target for reducing adverse cardiometabolic health risks [15]. Secondary outcomes include optimisation of, fasting blood glucose, total cholesterol, HDL, LDL, triglycerides, body composition, food literacy, PA, and fruit and vegetable intake.

### Sample size calculation

Sample size calculation is based on waist circumference.

To calculate the sample size, we used the formula described by Rutterford, Copas [40], Table 3.

**Table 3:** Description of sample size calculation

$$\boldsymbol{m}=\frac{{\left({\boldsymbol{Z}}_{\mathbf{1}-\boldsymbol{\alpha} /\mathbf{2}}+{\boldsymbol{Z}}_{\mathbf{1}-\boldsymbol{\beta}}\right)}^{\mathbf{2}}\ \mathbf{2}{\boldsymbol{\sigma}}^{\mathbf{2}}}{{\boldsymbol{\Delta}}^{\mathbf{2}}}\left(\mathbf{1}+\left(\boldsymbol{n}-\mathbf{1}\right)\boldsymbol{\rho} \right)$$	
where? m = sample size per study arm	= 1.96 at the type 1 error of 5% = 0.842 at 80% power
σ = the standard deviation of primary outcome in the population	• Based on Uganda STEP survey [2], the standard deviation of waist circumference for women aged 18 to 49 years = 13.73.
△ = effect size = 7.4cm.	• Effect size was calculated based on Bendall, Mayr [41] , impact of the Mediterranean diet on central obesity. • Only studies with a duration below 12 months were included in the calculation as the proposed study duration is below 12 months.
(1 + (*n* − 1)*ρ*)= design effect (DE) to compensate for cluster randomisation. ▪ n = number of individuals per cluster ▪ ρ = Intra-Cluster Correlation coefficient	• For our intervention, n = 27 • ρ = 0.001 ▪ There is no data available on the intra-Cluster Correlation coefficient of waist circumference across villages for communities within Kampala. A range of 0.001 to 0.10 is demonstrated as a cost - effective and safe choice to account for the variability.

m = (1.96 + 0.842)^2^ *2*13.73^2^/7.4^2^) * (1 + (27-1) *0.001)

m = 55 participants

To cover for dropouts, m is increased by 20%, to make 66 participants.

Hence, the project aims to include 132 women in the trial, 66 in the treatment arm and 66 in the control arm

### Randomization

The six sub parishes (clusters) will be listed alphabetically. A cluster randomization with a 1:1 allocation will then be applied to randomize the sub parishes to either the treatment or control arm. In the sub parishes, women group leaders and participants will be blinded about the study arms.

### Data collection

Table 4 gives an overview of the different measurements and time points during the study.

**Table 4:** Measurements and time points

Parameter	Measurement method	Measurement moment		
		**Baseline**	**Post -intervention (3 months)**	**Post follow -up (3 months)**
**Metabolic indicators**				
Fasting blood glucose	CardioChek plus	X	X	X
Total cholesterol		X	X	X
HDL cholesterol		X	X	X
Triglycerides		X	X	X
Blood pressure	Seca b12 (twice)	X	X	X
Body composition	Body-stat 1500 lite touch	X	X	X
**Anthropometric**				
Weight	Weighing scale (Seca 874 dr) (twice)	X	X	X
Height	Seca height board (twice)	X		
Waist circumference	Tape (twice)	X	X	X
**Fruit and vegetable intake, food literacy and physical activity**				
Fruit and vegetable intake	Fruit and vegetable intake screener adapted from NIH screeners (https://epi.grants.cancer.gov/diet/screeners/fruitveg/instrument.html) , Additional file 9 Food literacy questionnaire (developed by our research team for Ugandan context in consideration of Poelman, Dijkstra [29] and Vidgen HA [27]. We are currently validating the questionnaire, Additional file 10	X	X	X
Food literacy		X	X	X
Physical activity (self-reported)	Short version of International Physical Activity Questionnaire (IPAQ-SF)	X	X	X
**Process indictors**				
Adherence to the programme	Attendance list		X	X
**Socio-demographic data**	Questionnaire	X		

### Data analysis

Data will be analysed using R software. To evaluate the effects of the intervention, multilevel analysis will be used. Using this technique, regression coefficients will be adjusted for the clustering of observations within sub parishes. We will define two levels in our multi-level analysis: (1) participant and (2) sub parishes. Linear mixed effect models will be used to examine the effect of the intervention on each of the outcome values. All analyses will be performed according to the intention-to treat-principle [42]. To assess changes in metabolic health between the intervention and control groups, a linear mixed effect model will be built where “time” (end line measurement (M_2_) will be compared with base-line measurement (M_1_) and post-follow up measurement (M_3_)), treatment (and interaction of time and treatment) as well as age will be specified as fixed effects, and sub parishes and participants as random factors. For all linear mixed models, compatibility with mixed-model assumptions will be checked by inspection of residual plots and Q-Q plots. In the case of heteroscedastic residuals, data will be log transformed. Tukey or Benjamini–Hochberg procedures will be applied when performing post hoc analyses to further identify differences within treatments as well as between time points. Statistical outliers will be defined as any observation which has an absolute residual exceeding 3 times the residual standard deviation. p < 0.05 will be considered significant in all analyses.

## Discussion

The paper reports the systematic development of a food literacy and PA intervention to optimise metabolic health among WRA, a strategic target group for dietary and PA behaviour change in the SSA setting. The developed intervention is comprehensive as it is built from a combination of scientific evidence, community needs, expert opinion, and theoretical models. The intervention is composed of five interactive group sessions focused on increasing knowledge, skills, and self-efficacy to develop a lifelong healthy and gastronomic relationship with fruits, vegetables, and PA. The basis of the intervention is personalised goals and action plans tailored to personal metabolic health and lifestyle needs, and environmental opportunities. Topical evidence suggests that linking interventions to personal metabolic health and lifestyle needs, and environmental opportunities may help drive behaviour change and positively influence health outcomes [37]. While time consuming and labour intensive, the IM protocol ensured that our intervention is hinged on a clear behaviour change theory and BCTs, which increases the likelihood of enacting a positive behaviour change and implementation in practice [20, 43].

Our intervention has several unique features. First, to the best of our knowledge, this is the first rigorously designed combined dietary and PA intervention targeting WRA in urban SSA [16]. Currently, there is limited evidence on how to tackle the increasing burden of suboptimal metabolic health in urban SSA. Second, the intervention is developed basing on the contextual determinants. Scientific evidence highlights that successful health behavioural change interventions should be based on contextual determinants [20]. Our systematic review [16] and qualitative study [17] revealed unique determinants to this setting and recommended a need to address undesirable subjective norms, gaps in knowledge, skills, and self-efficacy. Accordingly, the intervention objectives are centred on the findings of systematic review [16] and qualitative study [17]. Third, the PO of the intervention were framed around the food literacy model, an effective holistic model. Food literacy focuses on a “how to do approach” in contrast to the ineffective traditional approaches of focusing on only nutrition knowledge [44]. Improving knowledge mainly creates an intention towards health behaviour. However, intentions rarely translate into actual behaviour” [45].

Fourth, the intervention is designed with a combination of eleven evidence based BCT, scientifically proven to enact changes in knowledge, skills, self-efficacy, subjective norms, and social support to create a sustainable behaviour change [20, 35, 36, 43]. The limitation is that the evidence for the effectiveness of the BCT is exclusively from HIC due to lack of evidence in urban SSA. Questions may be raised on the applicability and effectiveness of scientifically proven BCT in HIC in urban SSA. Theoretically, scientifically proven BCT in the HIC, could positively influence behaviour change in other settings provided they are translated into practical strategies with critical consideration of parameters of use documented by Eldredge, Markham [20]. In our intervention, we carefully considered the parameters of use suggested by Eldredge, Markham [20]. If successful, the intervention could contribute evidence on the validity/applicability of scientifically proven BCT in High Income Countries in urban SSA and how they can be translated.

Fifth, our intervention equips participants with skills and self-efficacy to evaluate nutrition information. Studies show that over the last decade, the virtual environment has become an important determinant of dietary and PA behaviour [46]. A lot of nutrition information is shared on social and mass media, yet majority of the public lacks the skill and self-efficacy to distinguish between factual and non-factual nutrition information. Lastly, in our evaluation design of the intervention, we have included a post intervention follow-up measurement moment. This allows us to analyse the long-lasting effect of the intervention.

Our intervention has some limitations. First, in our intervention design, we did not address the environmental determinants which our needs assessment found to have a significant influence on dietary and PA behaviour in urban Ugandan setting. For example, we did not directly influence food deserts and the increasingly sedentary physical environments (cheap motorised transport means). Our resources could not enable us to tackle the environmental determinants. Additionally, changes in food environment and transportation systems necessitates policy changes driven by multi sectoral government structures. However, through the food literacy model we equip participants with practical tips to negotiate the prevailing environment. Secondly, we did not create an intervention for adoption, implementation, and sustainability according to the principles of IM protocol. Creating an intervention for adoption and sustainability was beyond the scope of our resources and timeline. Hence, we adapted the IM protocol to focus on the development of the implementation plan to evaluate the developed behavioral change intervention through a proof-of-concept study. Accordingly, relevant potential environmental actors/implementers of the intervention in the community were not involved. This could create issues with implementation/institutionalization of our intervention later. However, we involved women group leaders within the religious institutions in the development of the intervention. They are as well foreseen to be involved in the execution of the proof concept intervention evaluation study. The involved women group leaders could be a starting point in designing the adoption, implementation and sustainability intervention if our proof concept evaluation study proves the intervention effectiveness.

## Conclusions

The paper describes the development of a combined food literacy and PA intervention. The developed intervention is an innovative approach as is it is created around food literacy. Additionally, the intervention is built on determinants specific to urban Uganda and evidence based behavioural change theoretical models and techniques, resulting in a clear mechanism of the hypothesised behavioural change. If the planned randomised control trial is found to improve the metabolic health among WRA, the developed intervention would be an important strategy in urban Uganda and SSA at large. The detailed description of our intervention can guide future intervention development in urban Uganda and SSA.

## Supplementary Information

**Additional file 1.**

**Additional file 2.**

**Additional file 3.**

**Additional file 4.**

**Additional file 5.**

**Additional file 6.**

**Additional file 7.**

**Additional file 8.**

**Additional file 9.**

**Additional file 10.**

## Data Availability

The datasets used and/or analysed during the current study are available from the corresponding author on reasonable request.

## Abbreviations

PA: physical activity
NCD: Non-Communicable Diseases
LMIC: Low- and Middle-Income Countries
WHO: World Health Organisation
SSA: sub-Saharan Africa
HIC: High Income Countries
WRA: women of reproductive age
IM: intervention mapping protocol
PO: performance objectives
FGD: focus group discussions
BCT: behavioural change techniques
