# Supplementary material for: The conceptual framework for a combined food literacy and physical activity intervention to optimize metabolic health among women of reproductive age in urban Uganda
Source: BMC Public Health. 2022 Feb 18;22:351. doi: 10.1186/s12889-022-12740-w (PMC8856934; doi:10.1186/s12889-022-12740-w)
Supplement: Supplementary file 1 — Additional file 1. [file 12889_2022_12740_MOESM1_ESM.docx]

**Additional file 1: Focus group discussions question guide (questions on implementation)**

1. Which organizations within community would you trust more to provide you with this information, probe for examples e. g churches, health centres.
2. How would you like to receive this information about how to eat healthy – probe for; organized group sessions, one on one session, cooking classes – healthy cooking skills, sharing information through social media. Why this selection?
3. At which venues would you like these activities to be held?
