# Supplementary material for: The conceptual framework for a combined food literacy and physical activity intervention to optimize metabolic health among women of reproductive age in urban Uganda
Source: BMC Public Health. 2022 Feb 18;22:351. doi: 10.1186/s12889-022-12740-w (PMC8856934; doi:10.1186/s12889-022-12740-w)
Supplement: Supplementary file 2 — Additional file 2. [file 12889_2022_12740_MOESM2_ESM.docx]

**Additional file 2**

**Table 1**: Methodological aspects of the studies conducted during intervention development and planned evaluation

|  | **Qualitative study (focus group discussion)(3)** | **Systematic review(8)** | **Intervention development (Step 2 to 5)** | **Planned evaluation study** |
| --- | --- | --- | --- | --- |
| Study setting | Kampala, Uganda | According to PRISMA guidelines | Kampala, Uganda | Kampala, Uganda |
| Participants | Women of reproductive age (18 to 45 years) |  | Executed by planning group, divided into two small groups  **Planning group 1**: Professors with expertise in behavioral nutrition and PA (n=4), a PhD researcher and MSc. Human Nutrition students (n=4)  **Planning group 2:**  Female representatives from the Institutional religious women groups (Mbuya Catholic Parish) (n=4). Purposively selected 4 women leaders who had participated in the qualitative study **(3)** | Women of reproductive age (18 to 45 years) |
| Recruitment sites | Institutional religious women groups (Mbuya Catholic Parish and Beauty for Ashes Ministries) |  |  | Institutional religious women groups (Mbuya Catholic Parish – six sub parishes to act as clusters) |
| Sampling strategy | Purposive sampling |  |  | Cluster randomization with a 1:1 allocation |
| Inclusion/exclusion criteria | Gender (women)  Age (18–45 years). No exclusion was done based on health conditions or anthropometric indices |  |  | Inclusion   - Sex (women), - Age (18 to 45 years), - Central obesity [waist circumference ≥ 80 cm]), - Fluent in either Luganda or English (sessions will be conducted in Luganda/English) - Willingness to follow the three-months intervention and three months follow-up, - Willingness to sign the informed consent.   Exclusion criteria   - Being treated for diabetes Mellitus Type 1 or Type 2, hypertension, high cholesterol, or any other cardio-metabolic related disease. - Pregnancy. |
| Sample size | Data saturation - 12 focus group discussions, group size;5 to 8 participants |  |  | 132 participants (66 per study arm)  Calculated according to the formula described by Rutterford, Copas (10) |
| Date of study | Jan/Feb 2019 |  | June 2019 to October 2020 |  |
| Data analysis | Inductive thematic content analysis |  |  | Intention-to-treat analyses were performed using linear mixed models |
