# Supplementary material for: The conceptual framework for a combined food literacy and physical activity intervention to optimize metabolic health among women of reproductive age in urban Uganda
Source: BMC Public Health. 2022 Feb 18;22:351. doi: 10.1186/s12889-022-12740-w (PMC8856934; doi:10.1186/s12889-022-12740-w)
Supplement: Supplementary file 3 — Additional file 3. [file 12889_2022_12740_MOESM3_ESM.docx]

**Additional file 3: Matrices of change objectives**

**Table 1:** Matrices of change objectives for behavioral outcome 3 – women evaluate accuracy of food, nutrition, and PA information.

| **Performance objectives** | **Determinants** | | |
| --- | --- | --- | --- |
|  | **Knowledge** | **Skills** | **Self-efficacy** |
| **PO1:** Women search for nutrition and PA information. | **K1a**: Women state sources of reliable food, nutrition, and PA information | **SK1a**: Women demonstrate the ability to find reliable information | **SF1:** Women express confidence to find reliable information. |
| **PO2:** Women judge the accuracy/correctness of food, nutrition, and PA information | **K2b:** Women state features which qualifies information to be credible or incredible. | **SK1b**: Women demonstrate the ability to apply criteria of what distinguishes facts from nonfactual information on trending nutrition information/to identify credible sources of information. | **SF2**: Women express confidence to judge accuracy of information. |

**Table 2:** Matrix of change objectives for behavioural objective 2. women engage in moderate intensity PA for at least 150 minutes a week

| **Performance objectives (PO)** | **Knowledge** | **Skills/self-efficacy** | **Subjective norm** | **Social support** |
| --- | --- | --- | --- | --- |
| **PO1:** Women plan specific moderate intensity PA moment in their daily schedule | **K1a**: Women state health and social benefits of PA  **K1b:** Women describe the different types of PA.  **K1c:** Women summarize the daily and weekly PA recommended guidelines for the different moderate intensity PA types.  **K1d:** Women state importance of having weekly PA plans | **SK1a:** Women demonstrate ability/need to do a self-assessment in of their PA levels in relation to the recommended levels to identify personal PA gaps.  **SK1b**: Women demonstrate the ability to do a self-inventory of their home, work, neighborhood, and community environment to identify potential PA opportunities available to them.  **SF1a**: Women recognize/identify health/social issues linked to lifestyle PA they are struggling with/they would like to prevent.  **SK1c:** Women select potential ways to increase PA within their schedule from on inventory of available PA opportunities.  **SK1d:** Women make PA plans fitting within their schedule in line with available PA opportunities.  **SF1b**: Women identify potential barriers to attaining PA plans and ways how to cope with the barriers. | **SN1a**: Women list other participants or role models from social environment who are routinely engaging in moderate PA.  **SN1b:** Women perceive that peer regardless of social class are increasing their moderate intensity PA levels.  **SN1c:** Women perceive that the cultural misconceptions regarding participation in moderate intensity PA are unfounded | **SP1a**: Women explain why social support is important in increasing and maintaining PA. |
| **PO2:** Women execute the planned specific moderate PA moment in their daily schedule |  | **SK2a:** Women evaluate their PA plans.  **SF2a**: Women identify potential barriers to attaining PA plans.  **SF2b:** Women describe/state practical tips to cope or overcome potential barriers to attaining PA plans. |  | **SP2a:** Women identify social buddies (with almost similar PA plans) from the group.  **SP2b:** Women with their social buddies synchronize their action plans |
| **PO3:** Women maintain the newly incorporated moderate intensity PA moment in their daily schedule |  | **SK3a**: Women demonstrate how to execute their PA plans and adapt plans when experiencing identified barriers accordingly.  **SF3a**: Women recognize that evaluation of execution of implementation of action plan will result in practical solutions to encountered barriers, resulting in a sustained behavioral change |  | **SP3a**: women review each other’s implementation plans and adapt plans when experiencing barriers |

**Table 3:** Matrices of change objectives for behavioral outcome 3: Women consume at least one portions of vegetables and one portion of fruit every day.

| **Performance objective** | **Behavioral determinants** | | | |
| --- | --- | --- | --- | --- |
|  | **Knowledge** | **Skills and self-efficacy** | **Subjective norm/social support** | |
| **PO1:** Women decide/plan to eat more fruits and vegetables | **K1a**: Women summarize healthy eating – food group concept (with emphasis on fruits & vegetables)  **K1b:** Women summarize recommended intakes for fruit and vegetable (WHO)  **K1d:** Women list the benefits (health & social) of eating fruits?  **K1e:** Women list the benefits (health & social) of eating vegetables?  **K1f**: Women describe why we need both fruits and vegetables (one cannot substitute another)  **K1g:** Women know the importance of variation towards realizing health benefits and gastronomy | **SF1a:** Women recognize need to increase fruit and vegetable intake based on current intake in relation to recommended levels.  **SF1b**: Women recognize that healthy eating is not introducing totally new foods to current diets but a modification (few shifts in current dietary pattern).  **SF1c**: Women recognize that increasing fruit and vegetable consumption can have positive health (NCD) effects.  **SF1d**: Women anticipate and identify barriers to increasing vegetable consumption.  **SF1e**: Women anticipate and identify barriers to increasing fruit consumption.  **SF1f**: Women explain practical tips to cope or overcome barriers limiting increasing vegetable/fruit consumption.  **SF1g**: Women explain practical tips to cope or overcome barriers limiting increasing fruit consumption | **SN1a**: Women list other participants or role models from social environment who are eating more fruits and vegetables.  **SN1b**: Women perceive that peer regardless of social class are increasing intake of fruits and vegetables | |
| **PO2:** Women buy fruits and vegetables | **K2a:**  Women list at least three relevant aspects of food shopping planning  **K2b:** Women list commonly available vegetables  **K2c**: Women describe at least 3 favorite vegetables in season.  **K2d**: Women list commonly available fruits  **K2e:** Women from the list-find at least 3 favorite fruits in season  **K2f:** Women identify affordable sources of quality (safe) fruits and vegetable foods within neighborhood.  **K2g**: Women identify fruits and vegetables to buy in large amounts based on storage space and facilities.  **K2h**: Women describe quality aspects to note when buying fruits and vegetables | **SF2a:** Women recognize that it is possible to eat tasty meals containing vegetables and fruits on budget and time while still choosing according to preferences.  **SK2a:** Women demonstrate ability to come up with weekly meal plans containing at least one portion of a fruit on a daily basis.  **SK2b:** Women demonstrate ability to come up with weekly meal plans containing at least one portions of vegetables on a daily basis.  **SF2b**: Women anticipate and identify barriers to attaining weekly meal plans containing at least one portion of vegetables and ways to cope or overcome the identified barriers.  **SF2c**: Women anticipate and identify barriers to attaining weekly meal plans containing at least two portions of fruits and ways to cope or overcome the identified barriers | **SN2a**: Women perceive that peer regardless of social class people buy fruits and vegetables following the demonstrated practical tips. | |
| **PO3:** Women prepare family meals rich in vegetables and fruits | **K3a**: Women state at least three ways to reduce pesticide residues on fruits and vegetables.  **K3b:** Women state at least four cooking methods to cook tasty vegetables.  **K3c:** Women state at least 3 advantages and 3 disadvantages of each of the stated preparation/cooking methods | **SK3a:** Women demonstrate basic stepwise preparation techniques to reduce pesticide residues on fruits and vegetables.  **SK3b**: Women demonstrate practical ways to cope with cumbersome work associated with vegetables pre-preparation.  **SK3c:** Women identify and demonstrate ways to cook healthy tasty vegetables.  **SF3a**: Women expect to have safe fruits and vegetables hygienic and free of pesticide residues.  **SF3b**: Women expect to eat tasty vegetables | **SN3a**: Women perceive that peers prepare vegetables in the demonstrated practical ways. | |
| **PO4:** Women eat vegetables and fruits in varying environments (traveling, at work) |  | **SF4a**: Women anticipate and list barriers to eating vegetables in varying environments (when at work, traveling)?  **SK4a**: Women identify and demonstrate practical tips on how you can attain vegetable consumption across the day when at work, traveling.  **SF4b**: Women anticipate and list barriers to eating fruits in varying environments (when at work, traveling)?  **SK4b**: Women identify and demonstrate practical tips on how you can attain fruit consumption across the day when at work, traveling.  **SF4C**: Women recognize that with prior planning you can eat fruits and vegetables in every environment | **SN4a**: Women demonstrate that peers eat vegetables in the demonstrated practical ways.  **SN4b:** Women demonstrate that peers eat fruits in the demonstrated practical ways | |
| **PO5:** Women maintain newly learned buying, cooking, and eating habits? |  | **SK5a:** Women demonstrate ability to evaluate execution of their fruit/veg action plans and adapt their goals accordingly.  **SF5a**: Women recognize that evaluation of execution of implementation of action plan will result in practical solutions to encountered barriers, resulting in a sustained behavioral change | **SPa:** Group members demonstrate ability to review each other’s implementation plans and offer support to each other |  |
