# Supplementary material for: The conceptual framework for a combined food literacy and physical activity intervention to optimize metabolic health among women of reproductive age in urban Uganda
Source: BMC Public Health. 2022 Feb 18;22:351. doi: 10.1186/s12889-022-12740-w (PMC8856934; doi:10.1186/s12889-022-12740-w)
Supplement: Supplementary file 4 — Additional file 4. [file 12889_2022_12740_MOESM4_ESM.docx]

**Additional file 4:** Matrices of BCT and practical strategies

**Table 1:** change methods and practical strategies. women evaluate accuracy food, nutrition, and PA information.

| **Performance objective** | **Determinants** | **Change objectives** | **Change methods** | **Parameters of use** | **Practical applications** |
| --- | --- | --- | --- | --- | --- |
| **PO1: Women search for food, nutrition, and PA information.** | **Knowledge** | **K1a**: Women state sources of reliable food, nutrition, and PA information | Information via imagery | Imagery – infographics used draw examples from Ugandan setting. | With aid of infographics, in a group session, moderator notes out key sources for of reliable food, nutrition and PA information |
|  | **Skills** | **SK1a:** Women demonstrate the ability to find reliable information | Motivational interviewing  Guided practice | Interactive group sessions where participants are encouraged to identify problems and suggest solutions. | In a group session, applying quality criteria (key features of credible info), a moderator guides participant to identify credible sources of information.  Moderator asks each participant to come up with a question on nutrition tip they would like to know more about. Using the UK NHS database, trainer guides participants to find answer to one of the questions. |
|  | **Self-efficacy** | **SF1**: Women express confidence to find reliable information | Guided practice |  | In a group session, applying quality criteria (key features of credible info), a moderator guides participant to identify credible sources of information.  Moderator asks each participant to come up with a question on nutrition tip they would like to know more about. Using the UK NHS database, trainer guides participants to find answer to one of the questions. |
| **PO2: Women judge the accuracy/correctness of food, nutrition, and PA information** | **Knowledge** | **K1a:** Women state features which qualifies information to be credible or incredible. | Information via imagery | Imagery – infographics used draw examples from Ugandan setting. | With aid of infographics, in a group session, moderator notes out key features to use to assess reliability of information |
|  | **Skills** | **SK1a:** Women demonstrate the ability to apply criteria of what distinguishes facts from nonfactual information on trending nutrition information/to identify credible sources of information**.** | Motivational interviewing  Guided practice | Interactive group sessions where participants are encouraged to identify problems and suggest solutions. | In a group session, using an example of trending nutrition information, a moderator guides participant to evaluate the information using the quality criteria. |
|  | **Self-efficacy** | **SF2**: Women express confidence to judge accuracy of information. | Guided practice |  | With their social buddies, participants find a nutrition information article, and assess its credibility using the provided quality criteria. They then discuss their conclusions to the entire group. |

**Table 2:** Change methods and practical strategies: Women engage in moderate intensity PA for at least 150 minutes a week.

| **Performance objective** | **Determinants** | **Change objectives** | **Change methods** | **Parameters of use** | **Practical applications** |
| --- | --- | --- | --- | --- | --- |
| **PO1: Women plan specific moderate intensity PA moment in their daily schedule** | **Knowledge** | **K1a**: Women state health and social benefits of PA  **K1b:** Women describe the different types of PA.  **K1c:** Women summarize the daily and weekly PA recommended guidelines for the different moderate intensity PA types.  **K1d:** Women state importance of having weekly PA plans | Providing information through Imagery      Motivation interviewing | Imagery – infographics used draw examples from Ugandan setting.  Interactive group sessions where participants are encouraged to identify problems and suggest solutions. | Infographics (based on WHO PA triangle) on benefits/recommendations/practical tips to increase PA.  Interactive group sessions discuss major health issues in Kampala, |
|  | **Skills** | **SK1a:** Women demonstrate ability/need to do a self-assessment in of their PA levels in relation to the recommended levels to identify personal PA gaps.  **SK1b**: Women demonstrate the ability to do a self-inventory of their home, work, neighbourhood, and community environment to identify potential PA opportunities available to them.  **SK1c:** Women select potential ways to increase PA within their schedule from on inventory of available PA opportunities.  **SK1d:** Women make PA plans fitting within their schedule in line with available PA opportunities. | Motivation interviewing  Implementation intentions  Prompt specific goal setting  Action planning | Interactive one on one sessions where participants are encouraged to take the lead.  Existing positive intention (motivational interviewing & information provided in PO1)  Skills available as practical tips to increase PA are provided. | Interactive group sessions with one-on-one moments to discuss personal metabolic health indicator results following assessment.  Considering their daily routines, using a self-assessment tool, participants collect information.   - On their current PA levels - Data is used to create a personal PA profile. Personal PA profile is compared to PA recommendations to identify needs   Using a self-inventory tool, participants identify available potential PA opportunities within their environment and routine.  Creating “if then plans” in line with metabolic health and PA needs.  SMART PA goals, detailed action plans to achieve set SMART goals drawn considering environmental opportunities. |
|  | **Self-efficacy** | **SF1a**: Women recognize/identify health/social issues linked to lifestyle PA they are struggling with/they would like to prevent.  **SF1b**: Women identify potential barriers to attaining PA plans and ways how to cope with the barriers. | - Planning coping responses | Interactive feedback group sessions aid participants to identify barriers and potential solutions (coping responses) to carry out. | - Participants write down at least two things in their daily lives that could be a barrier to reaching their PA goal.   A moderator discusses with participants potential solutions (pre-defined list of practical tips). Interactive group sessions to come up with more solutions |
|  | **Subjective norms/social support** | **SN1a**: Women list other participants or role models from social environment who are routinely engaging in moderate PA.  **SN1b:** Women perceive that peer regardless of social class are increasing their moderate intensity PA levels.  **SN1c:** Women perceive that the cultural misconceptions regarding participation in moderate intensity PA are unfounded.  **SP2a**: Women explain why social support is important in increasing and maintaining PA. | Role modeling  Social support through exchanging ideas | Positive deviant group member – resembles and participants identify with her.  Aalready existing group – thus know and care for each other. | Interactive group sessions and positive deviant led feedback interactive group talks |
|  |  |  |  |  |  |
| **PO2:** **Women execute the planned specific moderate intensity PA moment in their daily schedule** | **Skills/self-efficacy** | **SK2a:** Women evaluate their PA plans. | - Self-monitoring | Goal progress reviewed. Potential barriers and solutions are identified to encourage participants. At the 3 months & 6 months metabolic health assessments and participants visualize their progress | - A tool to keep PA consumption records. |
|  |  | **SF2a**: Women identify potential barriers to attaining PA plans.  **SF2b:** Women come up with practical tips to cope or overcome potential barriers to attaining PA plans. | - Planning coping responses | Interactive feedback group sessions aid participants to identify barriers and potential solutions (coping responses) to carry out. | - Participants write down at least two things in their daily lives that could be a barrier to reaching their PA goal. - A moderator discusses with participants potential solutions (pre-defined list of practical tips). Interactive group sessions to come up with more solutions |
|  | **Social norms/social support** | **SP2a:** Women identify social buddies from the group.  **SP2b:** Women with their social buddies synchronize their action plans | - Social support through exchanging ideas | Already existing group – thus know and care for each other | - With guidance of trainer, group members identify PA buddies. criteria – members you spend with significant amount of time, you share a PA goal and implementation intentions |
| **PO3: Women maintain the newly incorporated moderate intensity PA moment in their daily schedule.** | **Skills/self-efficacy** | **SK3a**: Women describe demonstrate how to execute their PA plans and adapt plans when experiencing identified barriers accordingly.  **SF3a**: Women recognize that evaluation of execution of implementation of action plan will result in practical solutions to encountered barriers, resulting in a sustained behavioral change | Feedback on performance  Planning coping responses  Role modeling | Brief interactive group feedback sessions at the start of every session. One on one moments with quiet group members. | - Brief interactive group feedback sessions on goal progress, barriers & solutions. Goal review sessions (interactive group sessions with moments of one on one with the moderator) - Positive deviant led feedback interactive group talks |
|  | **Subjective norm/social support** | **SP3a**: women review each other’s implementation plans and adapt plans when experiencing barriers | Social support through exchanging ideas | Already existing group – thus know and care for each other | Positive deviant led feedback interactive group talks. Interactive group sessions with moments of one on one with the moderator |

**Table 3: change methods and practical strategies** – women consume at least two portions of vegetables and one portion of fruit every day.

| **Performance objective** | **Determinants** | **Change objectives** | **Change methods** | **Parameters of use** | **Practical applications** |
| --- | --- | --- | --- | --- | --- |
| **PO1**: Women decide to eat more fruits and vegetables | **Knowledge** | **K1a**: Women summarize healthy eating – food group concept (with emphasis on fruits & vegetables)  **K1b:** Women summarize recommended intakes for fruit and vegetable (WHO)  **K1d:** Women list the benefits (health & social) of eating fruits?  **K1e:** Women list the benefits (health & social) of eating vegetables?  **K1f**: Women describe why we need both fruits and vegetables (one cannot substitute another)  **K1g:** Women know the importance of variation towards realizing health benefits and gastronomy | - Providing information using Imagery - Motivation interviewing |  | Infographics on benefits/recommendations (healthy plate-based WHO guidelines) /practical tips to increase vegetable and fruit intake.  Interactive group sessions discuss major health issues in Kampala |
|  | **Skills and self-efficacy** | **SF1a:** Women recognize need to increase fruit and vegetable intake based on current intake in relation to recommended levels.  **SF1b**: Women recognize that healthy eating is not introducing totally new foods to current diets but a modification (few shifts in current dietary pattern).  **SF1c**: Women recognize that increasing fruit and vegetable consumption can have positive health (NCD) effects. | - Motivation interviewing |  | Interactive group sessions and one-on-one moments to discuss personal metabolic health indicator results following assessment.   - Participants compile information on their current fruit and vegetable intake. - Data is used to create a weekly personal fruit and vegetable intake profile. - Personal fruit and vegetable intake profile is compared to recommendations to identify needs.   Using a self-inventory tool, participants identify available potential opportunities within their environment  Interactive group sessions discuss major health issues in Kampala  Healthy plate-based WHO guidelines) /practical tips to increase vegetable and fruit intake. |
|  |  | **SF1b**: Women anticipate and identify barriers to increasing vegetable consumption | Motivation interviewing |  | - Interactive group sessions: participant brainstorm (speak up or write on flipchart) on barriers limiting intake of fruits/vegetables. |
|  |  | **SF1c**: Women anticipate and identify barriers to increasing fruit consumption |  |  |  |
|  |  | **SF1d**: Women explain practical tips to cope or overcome barriers limiting increasing vegetable/fruit consumption.  **SF1e**: Women explain practical tips to cope or overcome barriers limiting increasing fruit consumption | Motivation interviewing |  | - Interactive group sessions: Participants brainstorm solutions to barriers limiting intake of fruits/ vegetables. - Infographics on practical tips to increase consumption of fruits and vegetables |
|  | **Subjective norm** | **SN1a**: Women list other participants or role models from social environment who are eating more fruits and vegetables.  **SN1b**: Women perceive that peer regardless of social class are increasing intake of fruits and vegetables | Role modeling |  | Positive deviant led feedback interactive group talks |
| **PO2**: Women buy and incorporate fruits and vegetables in their meal plans | **Knowledge** | **K2a:**  Women list at least three relevant aspects of food shopping planning  **K2b:** Women list commonly available vegetables  **K2c**: Women describe at least 3 favorite vegetables in season.  **K2d**: Women list commonly available fruits  **K2e:** Women from the list-find at least 3 favorite fruits in season  **K2f:** Women identify affordable sources of quality (safe) fruits and vegetable foods within neighborhood.  **K2g**: Women identify fruits and vegetables to buy in large amounts based on storage space and facilities.  **K2h**: Women describe quality aspects to note when buying fruits and vegetables | - Motivation interviewing - Providing information through imagery |  | Interactive group sessions on importance of planning, and practical tips to eating more fruits and vegetables in varying environments.  Infographics locally available fruits/vegetables on practical tips to increase consumption of fruits and vegetables |
|  | **Skills and self-efficacy** | **SF2a:** Women recognize that it is possible to eat tasty meals containing vegetables and fruits on budget and time while still choosing according to preferences.  **SK2a:** Women demonstrate ability to come up with weekly meal plans containing at least one portion of a fruit on a daily basis.  **SK2b:** Women demonstrate ability to come up with weekly meal plans containing at least one portions of vegetables on a daily basis.  **SF2b**: Women anticipate and identify barriers to attaining weekly meal plans containing at least one portion of vegetables and ways to cope or overcome the identified barriers.  **SF2c**: Women anticipate and identify barriers to attaining weekly meal plans containing at least two portions of fruits and ways to cope or overcome the identified barriers | - Motivation interviewing - Implementation intentions - Prompt specific goal setting. - Action planning - Planning coping responses - Self-monitoring |  | - Creating “if then plans” in line with metabolic health and PA needs. - SMART fruit/vegetable goals, detailed action plans to achieve set SMART goals drawn considering environmental opportunities. - A tool to keep fruit/vegetable consumption records. |
|  | **Subjective norm** | **SN2a**: Women perceive that peer regardless of social class people buy fruits and vegetables following the demonstrated practical tips. | Role modeling |  | Positive deviant led feedback interactive group talks |
| Women prepare family meals rich in vegetables and fruits | **Knowledge** | **K3a**: Women state at least three ways to reduce pesticide residues on fruits and vegetables.  **K3b:** Women state at least four cooking methods to cook tasty vegetables.  **K3c:** Women state at least 3 advantages and 3 disadvantages of each of the stated preparation/cooking methods | - Motivation interviewing - Providing information through imagery |  | - Interactive group sessions - brainstorm (write on flip chart or speak up) a list of techniques for veg preparation methods, noting out advantages & disadvantages of each method. - Infographics of local vegetable recipes |
|  | **Skills and self-efficacy** | **SK3a:** Women demonstrate basic stepwise preparation techniques to reduce pesticide residues on fruits and vegetables.  **SK3b**: Women demonstrate practical ways to cope with cumbersome work associated with vegetables pre-preparation.  **SK3c:** Women identify and demonstrate ways to cook healthy tasty vegetables.  **SF3a**: Women expect to have safe fruits and vegetables hygienic and free of pesticide residues.  **SF3b**: Women expect to eat tasty vegetables | - Guided practice |  | Practical trial of local recipes in group cooking sessions |
|  | **Subjective norm** | **SN3a**: Women perceive that peers prepare vegetables in the demonstrated practical ways | - Role modeling |  | Interactive group sessions/ feedback during cooking sessions |
|  |  |  |  |  |  |
| **PO4:** Women eat vegetables and fruits in varying environments (at work) | **Skills and self-efficacy** | **SF4a**: Women anticipate and list barriers to eating vegetables in varying environments (when at work, traveling)?  **SK4a**: Women identify and demonstrate practical tips on how you can attain vegetable consumption across the day when at work, traveling.  **SF4b**: Women anticipate and list barriers to eating fruits in varying environments (when at work, traveling)?  **SK4b**: Women identify and demonstrate practical tips on how you can attain fruit consumption across the day when at work, traveling.  **SF4C**: Women recognize that with prior planning you can eat fruits and vegetables in every environment | Motivation interviewing |  | Interactive group sessions where barriers to consumption of fruits and vegetables in different environments are discussed.  Practical tips (solutions) presented in form of infographics are discussed |
|  | **Subjective norm** | **SN4a**: Women demonstrate that peers eat vegetables in the demonstrated practical ways.  **SN4b:** Women demonstrate that peers eat fruits in the demonstrated practical ways | - Motivation interviewing - Role modeling |  | Positive deviant led feedback interactive group talks |
| **PO5:** Women maintain newly learned buying and cooking habits? | **Skills and self-efficacy**    **Social support** | **SK5a:** Women demonstrate ability to evaluate execution of their fruit/veg action plans and adapt their goals accordingly.  **SF5a**: Women recognize that evaluation of execution of implementation of action plan will result in practical solutions to encountered barriers, resulting in a sustained behavioral change  **SPa:** Group members review each other’s implementation plans and offer support to each other | - Planning coping responses - Feedback - Role modelling - Social support through exchanging ideas |  | Brief interactive group feedback sessions on goal progress, barriers & solutions. Goal review sessions (interactive group sessions with moments of one on one with the moderator)  Positive deviant led feedback interactive group talks |
