# Supplementary material for: The conceptual framework for a combined food literacy and physical activity intervention to optimize metabolic health among women of reproductive age in urban Uganda
Source: BMC Public Health. 2022 Feb 18;22:351. doi: 10.1186/s12889-022-12740-w (PMC8856934; doi:10.1186/s12889-022-12740-w)
Supplement: Supplementary file 5 — Additional file 5. [file 12889_2022_12740_MOESM5_ESM.docx]

**Additional file 5**: Scope and sequence of the intervention

**Developed intervention – scope and sequence of the group sessions*.***

| **Session /timeline** | **Focus of session** | **Role of moderator + 2 assistants** | **Role of participants** | **Outcome** |
| --- | --- | --- | --- | --- |
| **Measurement moment 1** (**week 1**)  (PA & Diet) | Assessments and introduction of programme | - Introduction of programme - Establish group rules. - Take baseline biochemical measures (**lab technician**) – explain cut-off values (meaning of results - on a personalised basis). - Guide participants through PA assessment questionnaire (**work with two assistants**) - Guide participants through food literacy questionnaire (**work with two assistants**) | - Baseline biochemical measures (fasting glucose, lipid profile, body composition, blood pressure), waist circumference and BMI (weight and height) - Participants complete PA assessment tool to create personal physical activity profile - Participants complete food literacy questionnaire - Participants complete food environment questionnaire | Highlight personal needs & focus program planning |
| **Session 1** **(week** 1)  **Part 1 - 30 minutes (PA & diet)** | Health risk assessment and benefits of PA & healthy diet (focus on fruits & vegetables) | - Lead group discussion + brainstorming - What are the major health issues in Kampala now? - After participants brain storming, emphasis the NCD - What can be done to prevent these health issues? - After discussion, moderator points at importance of healthy diet (fruits & vegs as basis) and physical activity (- point out importance of combined effect of diet & PA)   **Introduce a 10 minutes break in form of exercise** | - Participants speak out or write out health issues they think on a flip chart - Participants reflect on the implications of their biochemical measure status - Participants suggest preventive measures to the raised health issues (speak out or write on a flip chart) - Do exercises (in bout of 10 minutes) with a fun factor (picked from contextualized physical activity triangle) e.g. - 5 Squats - Writing their first names using body movements - Sitting/standing up concurrently for 10 rounds - Stretching | - Participants think about their current metabolic health status & importance of maintaining it within normal levels. - Participants appreciate importance of fruits & vegs, and PA - Active relaxing (participants get practical tips of relaxing actively) |
| **Part 2 – 20 minutes**  Focus on PA | Types of PA and recommendations | **Lead group discussion**   - Using contextualised PA triangle demonstrate PA which improves heart rate and those which give muscle strengthening health benefits - Using contextualised PA triangle illustrate the PA recommendations |  | Increase participants knowledge of different ways to be physically active |
|  | Compare PA profile to recommendations | - Guide participants to compare personal PA profile to WHO recommendations over a typical week (compile results from baseline and project them to show gaps – then guide each participant to visualize personal gaps) - Brainstorm barriers/solutions to PA gaps | - **Participants** compare PA profile to recommendations to identify need to improve PA profile | Participants visualise their PA gaps |
| **Part 3 –** **60 minutes**  Focus on PA | Planning & setting weekly PA goals | Using an example, explain how to set SMART goals | - Participants create IF – then PA plans in line with personal biochemical measures and PA gaps - Participants identify & select available potential PA opportunities within their environment and routine - Participants set a weekly goal for cardiovascular exercises (work or travel related) and a weekly goal for muscle strengthening exercise (can be a group activity on a Saturday or intergenerational PA to be done with children and husband) – using **a tool** (participants can refer to practical tips & potential PA opportunity tool) | Participants come up with SMART PA goals and detailed plans to achieve to the goals |
| **Session 2 (week 2)** | Feedback session on PA goals (15 minutes) | Lead brief feedback session on progress of set PA goals (15 minutes) | Participants give an update on the progress of their goals (share enabling factors and challenges). | Solutions to challenges impeding achievement of set goals |
| **Part 1 - Focus on diet (20 minutes)** | Fruit & vegetable recommendations | **Lead group discussion**  Using a contextualized food plate visual (summary of WHO healthy eating guidelines):   - summarize the fruit/veg daily recommendations (emphasize that basis of healthy eating as vegs and fruits). Include what is meant by portion size. - why we need both fruits and vegetables - one cannot substitute another   importance of variation in terms of gastronomy & health | - Participants compare their current veg consumption to recommendations to identify need to improve veg consumption - Participants compare their current fruit consumption to recommendations to identify need to improve fruit consumption - Following example, participants follow healthy food plate guidelines to add fruits and vegs to their usual meals across a typical day (arbitrary example) | Increase participants knowledge of fruits & veg recommendations  Participants visualise their veg and fruit intake gaps |
| **Part 2 – focus on diet (20 minutes)** | Brainstorm barriers/solutions to limited intake of fruits and vegetables | **Lead group discussion + brainstorming**   - Pose a question; what the barriers/solutions to inadequate consumption of fruits and vegs | - Participants brainstorm (speak up or write on flipchart) on barriers limiting intake of fruits - Participants brainstorm on barriers (speak up or write on flipchart) limiting intake of vegetables - Participants brainstorm solutions to barriers limiting intake of fruits and vegetables | Participants visualize potential solutions to limited intake of fruits & vegs  Visualize limitations and opportunities to fruit and vegetable consumption within prevailing environment |
| **Part 3 – focus on diet (60 minutes)** | Planning & budgeting meals including vegs & fruits | - Introduce goal setting and meal planning/budgeting as a potential solution – Using an example, guide participants to come up with SMART goals, pointing at practical tips on ensuring consumption in varying environments | - Following an example, participants set SMART daily goals to eat atleast one portion of vegs (**guiding tool** – refer to practical tips on ensuring consumption in varying environments) - Participants set SMART daily goals to eat atleast one portion of fruit | Participants come up with SMART veg/fruit goals and detailed cost and time efficient plans to achieve to the goals |
| **Session 3 (week 3)**  **Part 1- Feedback session on progress of set goals (30 minutes)** | Feedback session on PA goals | Lead brief feedback session on PA goals (15 minutes) | - Participants give an update on the progress of their goals (share enabling factors and challenges). | Solutions to challenges impeding achievement of set goals |
|  | Feedback session on fruit & vegetable goals | Lead brief feedback session on fruit & vegetable goals (15 minutes) | - Participants give an update on the progress of their goals (share enabling factors and challenges). | Solutions to challenges impeding achievement of set goals |
| **Session 3 (week 3)**  **Part 2 – 30 minutes (information evaluation)** | Evaluation of nutrition information | - Introduce nutrition information evaluation (notes out key features to use to assess reliability of information) - Recommend some credible sources of nutrition information (UK – NHS website) - **Introduce a 10-minute break – exercise bout** | - In a group session, using an example of trending nutrition information (selected from social media platform), guided by the moderator, participants evaluate the credibility of the information piece | Participants visualize and get acquainted with key features/sources of reliable/evidence-based nutrition information. |
| **Session 3 (week 3)** | Veg preparation techniques | - Introduce veg preparation tips as a potential solution to increasing veg consumption | - participants brainstorm (write on flip chart or speak up) a list of techniques for veg preparation methods, note out advantages & disadvantages of each method |  |
| **Part 3 (60 minutes)**  Focus on diet | Veg preparation recipe trail | - Introduce veg preparation recipes - Adapt veg goals to include – veg recipe trails | - Participants practically try out at least three of the 12 veg recipes (compiled from different parts of Uganda and adapted to health recommendations). Recipes also include practical tips on how to reduce pesticide residues on vegs, and pre-preparation methods to address cumbersome work associated with pre-preparation of vegs. A volunteer participant leads the practical sessions. - Add recipe trials to their weekly goals | Increase veg preparation skills & self-efficacy (participants appreciate that it is possible to prepare tasty vegs within minimal time) |
| **Session 4 (week 4)**  **Part 1 Focus on PA (30 minutes)** | Review and adaptation of PA goals | - Guide participants to review their PA goals/plans (feedback sessions)      - An intervention champion (positive deviant from the group – those participants who accomplish their set goals successfully) gives a 5 -minute talk on her journey to being physically active – focus on challenges (tight schedule, social norms & laziness) & how she overcame them | - Participants speak up or write out on a flip chart enabling factors/challenges they faced during implementation of their plans to attain planned PA goals. At least every participant gives one enabling factor and one challenge. - Participants listen to the role model talk - Through question and answer session, participants together with role model brainstorm solutions to the challenges faced - Participants re-adapt their goals (try out other activities / increase on duration of activities or maintain their goals but modify plans) | Increase problem solving ability and self-efficacy to faced challenges |
| **Session 4 (week 4)**  **Part 1 Focus on diet (30 minutes)** | Review and adaptation of diet – veg goals | A role model (positive deviant from the group - those participants who accomplish their set goals successfully) gives a 5-minute motivation talk on her journey to increase vegetable intake – focus on challenges (tight schedule, food deserts within prevailing environment and social norms) & how she overcame them  **Introduce a 10-minute break – exercise bout** | - Participants speak up or write out on a flip chart enabling factors/challenges they faced during implementation of their plans to attain planned veg intake goals. At least every participant gives one enabling factor and one challenge. - Participants listen to the role model talk - Through question and answer session, participants together with role model brainstorm solutions to the challenges faced - Participants re-adapt their goals (e.g. increase portion sizes or maintain goals but modify plans) | Increase problem solving ability and self-efficacy to faced challenges |
| **Part 2**  **Focus on diet (30 minutes)** | Review and adaptation of diet – fruit goals | A role model (positive deviant from the group) gives a 5-minute motivation talk on her journey to increase fruits intake – focus on challenges (tight schedule, food deserts within prevailing environment and social norms) & how she overcame them | - Participants speak up or write out on a flip chart enabling factors/challenges they faced during implementation of their plans to attain planned fruit intake goals. At least every participant gives one enabling factor and one challenge. - Participants listen to the role model talk - Through question and answer session, participants together with role model brainstorm solutions to the challenges faced - Participants re-adapt their goals (e.g. increase portion sizes or maintain goals but modify plans) | Increase problem solving ability and self-efficacy to faced challenges |
| **Session 5**  **(week 7)**  **Part 1 (30 minute) - focus on diet** | Review and adaptation of diet – fruit/veg goals | Moderator leads group discussion  **Introduce a 15-minute break – exercise bout – aerobic video** | - Participants speak up or write out on a flip chart enabling factors/challenges they faced during implementation of their plans to attain planned fruit/veg intake goals. - Participants brainstorm solutions to the challenges faced - Participants re-adapt their goals (e.g. increase portion sizes or modify plans) | Increase problem solving ability and self-efficacy to faced challenges |
| **Part 2 (30 minute) - focus on PA** | Review and adaptation of PA goals | Moderator leads group discussion | - Participants speak up or write out on a flip chart enabling factors/challenges they faced during implementation of their plans to attain PA goals - Participants brainstorm solutions to the challenges faced - Participants re-adapt their goals (try out other activities / increase on duration of activities) | Increase problem solving ability and self-efficacy to faced challenges |
| **Measurement moment 2 - end line (week 12)**  Focus on diet and PA | **12 - week re-assessment (end line)** | Same assignments as in week 1 | - End-line biochemical measures - Participants complete PA questionnaire to create personal physical activity profile - Participants complete food literacy questionnaire |  |
| **Measurement moment 3 – post follow up (week 24)**  Focus on diet and PA | 24 - week re-assessment (after 12-week follow-up period) | Same assignments as in week 1 | - Follow-up biochemical measures - Participants complete PA questionnaire - Participants complete food literacy questionnaire |  |
