# Supplementary figures and images for: The conceptual framework for a combined food literacy and physical activity intervention to optimize metabolic health among women of reproductive age in urban Uganda
Source: BMC Public Health. 2022 Feb 18;22:351. doi: 10.1186/s12889-022-12740-w (PMC8856934; doi:10.1186/s12889-022-12740-w)

**Additional file 6:** Developed infographics for the intervention


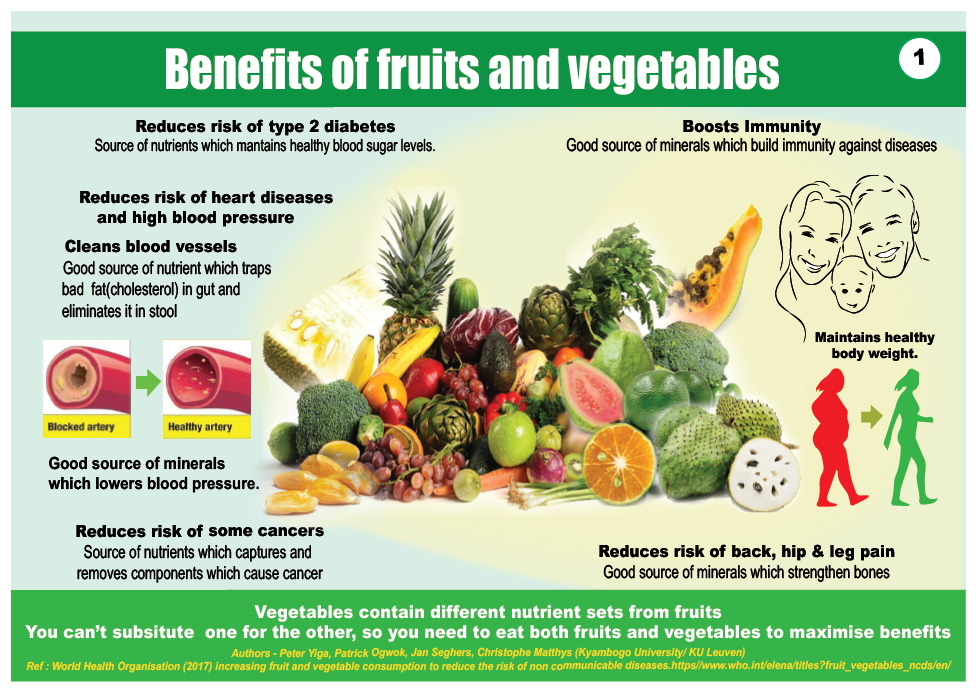


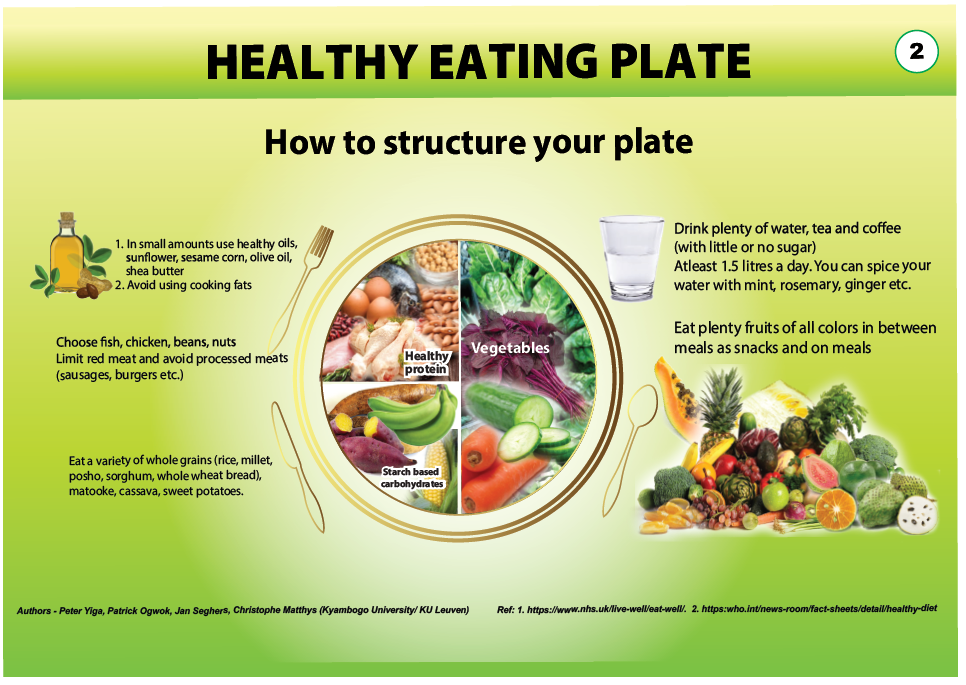


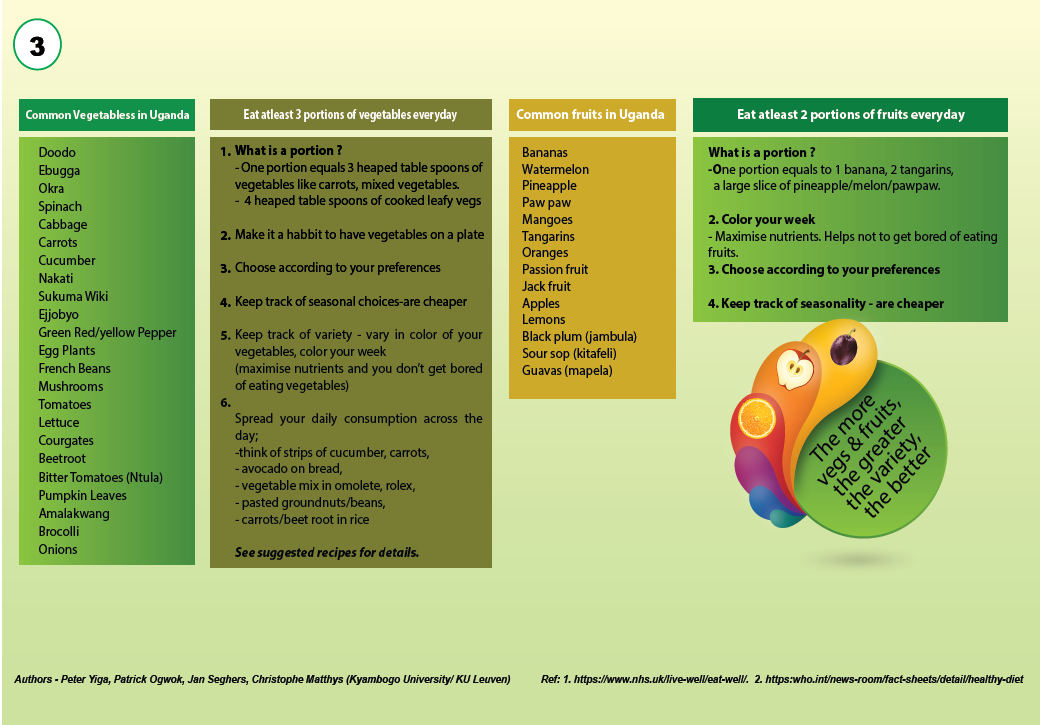


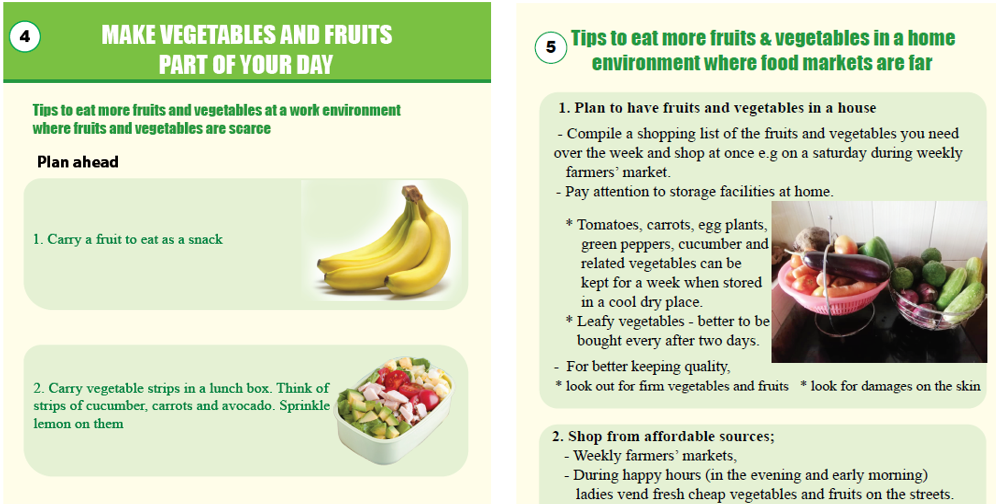


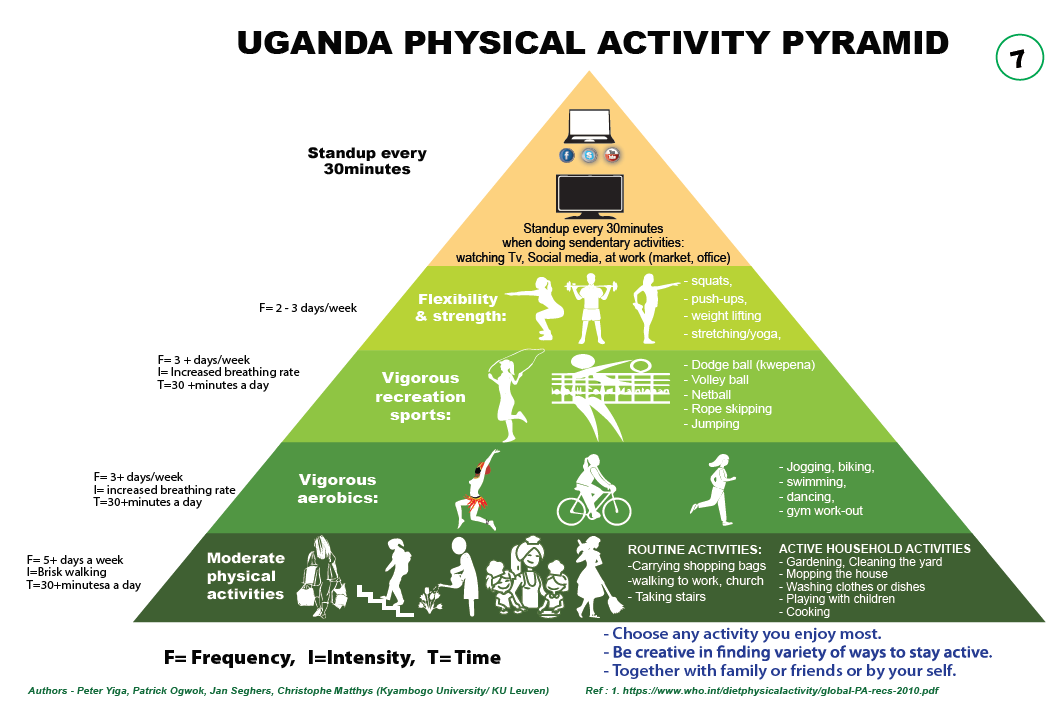


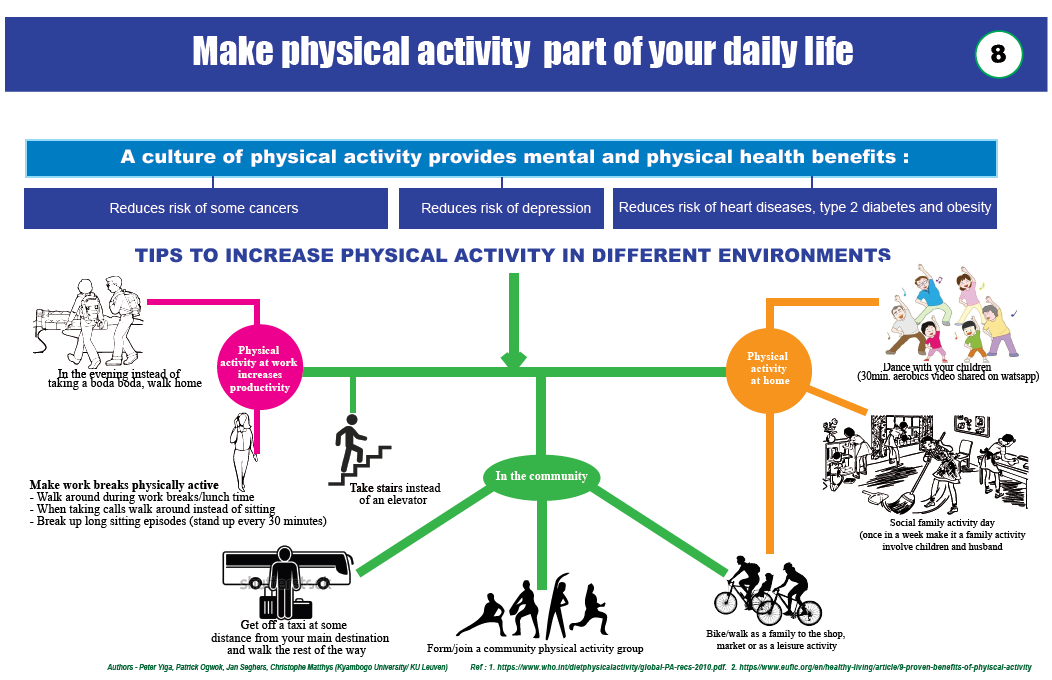


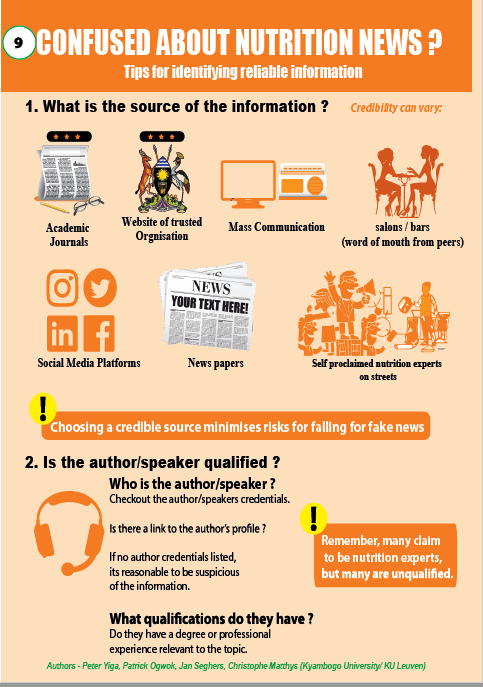

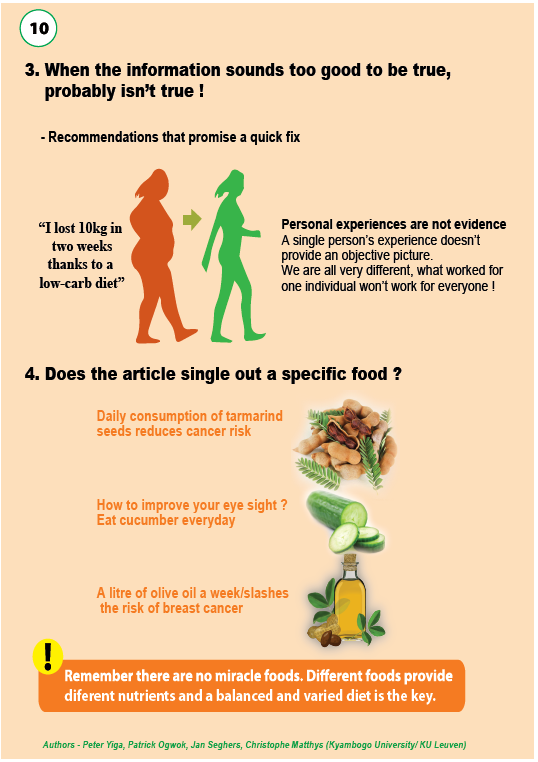


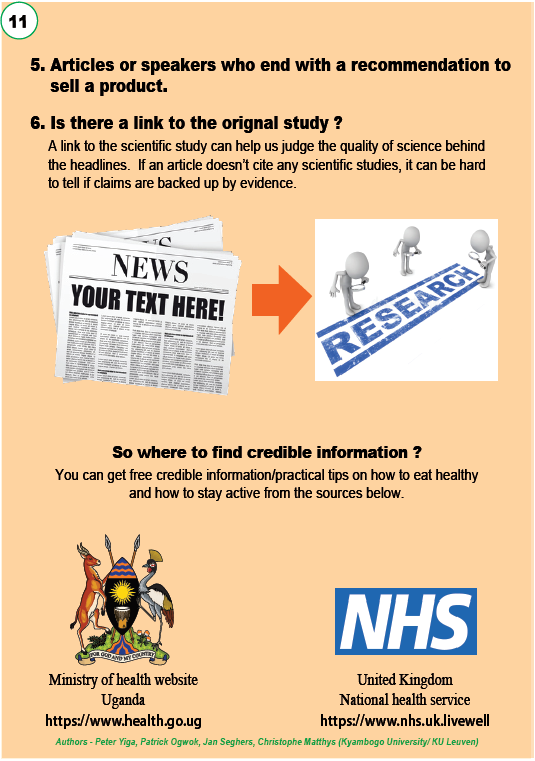

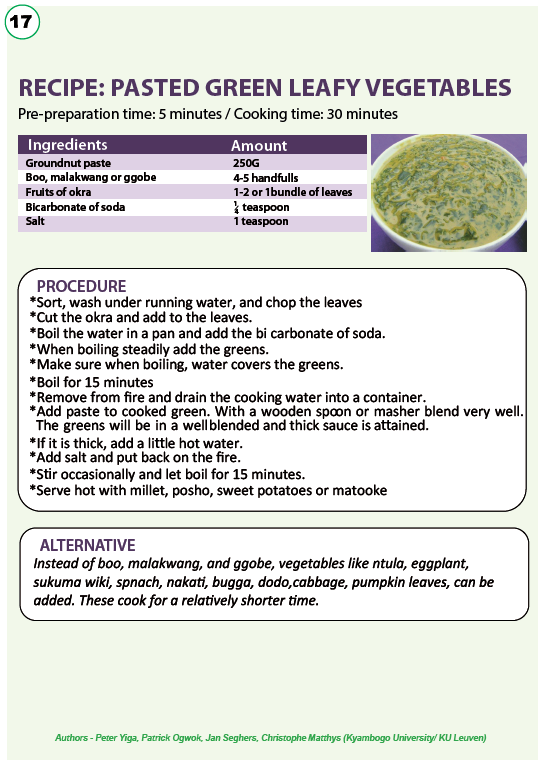


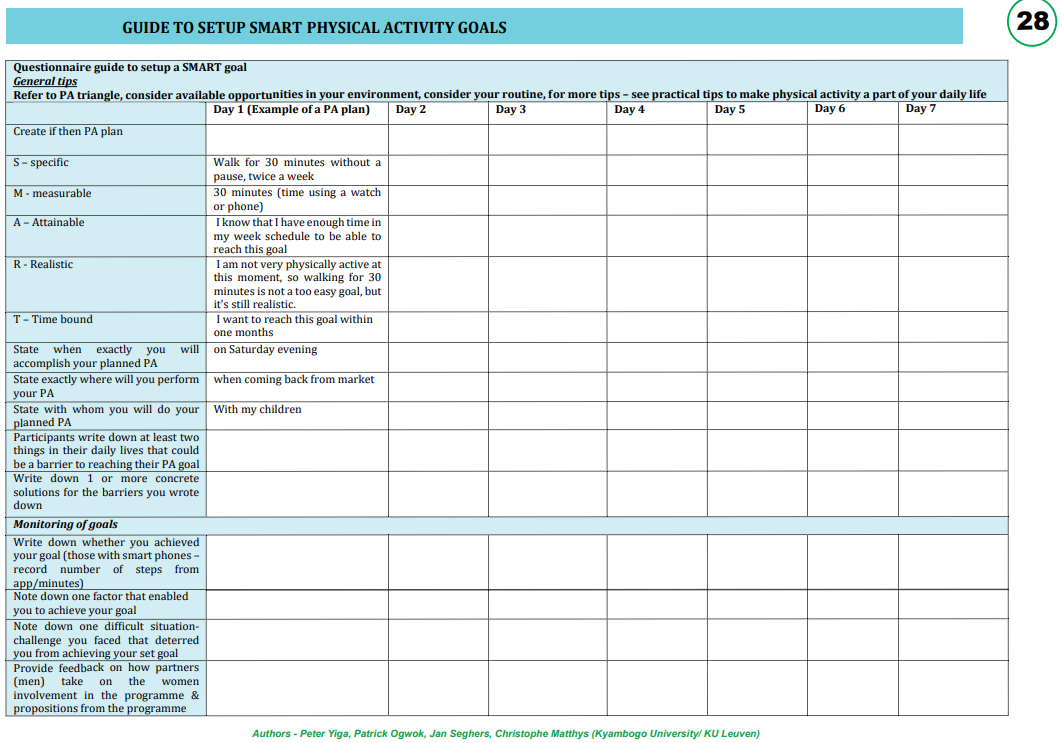

Supplement: Supplementary file 6 — Additional file 6. [file 12889_2022_12740_MOESM6_ESM.docx]
