# Supplementary material for: The conceptual framework for a combined food literacy and physical activity intervention to optimize metabolic health among women of reproductive age in urban Uganda
Source: BMC Public Health. 2022 Feb 18;22:351. doi: 10.1186/s12889-022-12740-w (PMC8856934; doi:10.1186/s12889-022-12740-w)
Supplement: Supplementary file 7 — Additional file 7. [file 12889_2022_12740_MOESM7_ESM.docx]

**Additional file 7:** Tools to assess PA and food environment for opportunities

Self-inventory tool to identify available potential PA opportunities within environment and routine

| **Home environment; please circle which items you have in your home** | | | |
| --- | --- | --- | --- |
| Bicycles | Backyard space | Sports shoes (running/ walking shoes) | Treadmills |
| Balls | Backyard garden | Sports wear | Tummy trimmer |
| Skipping ropes | Swings | Aerobic workout video tapes | Marts |
| Family members who frequently exercise or do related PA (husbands, other relatives) |  | Aerobic workout audio tapes |  |
| **Neighbourhood environment; please circle which of the following apply to your neighbourhood** | | | |
| Streetlights/security lights | Playgrounds | Children parks | Paved road sidewalks (to church, work and main markets) |
| Hills | Heavy traffic | Dogs that are unattended | Tarmacked roads (to church, work and main markets) |
| Marram un-dusty roads (to church, work and main markets) | Enjoyable beautiful scenery |  | Green open public parks |
| Swimming pool facility | Gym facility | Frequently see people walking (commuting or leisure) or exercising | Availability of social/community physical activity groups |
| **Work environment; please circle which of the following apply to your work environment** | | | |
| Cafeteria at a distance from office | Storied building (staircases) | Organized group workout sessions | Yard with green grass space (lawn) |
|  |  |  |  |
| **Convenient facilities; for each of these places where you can engage in exercise related PA, please indicate if it is on a frequently travelled route or within 20-minute walk from your home** | | | |
| Basketball court | Beach | Health spa or gym | Playground (netball, volleyball) |
| Aerobics centre | Green open public space | Swimming pool | Sports fields |
| On a scale of 5 what is the security of your neighbourhood | | |  |

**Food environment questionnaire to identify available potential opportunities within environment**

These questions explore your food environment.

This is not a test! There are no wrong answers. Just think about how you usually do things.

Please tick or circle in the box that provides the best answer for each question.

| **Home environment** | | | | | | |
| --- | --- | --- | --- | --- | --- | --- |
| 1 | Do you have a vegetable garden at home? | | | | | |
|  | 1: Yes | | | 2: No | | |
| 2 | Do you have a fruit garden/fruit trees at home? | | | | | |
|  | 1: Yes | | | 2: No | | |
| **Neighbourhood environment** | | | | | | |
| 3 | **Which of the food shopping possibilities do you consider having an affordable range of fruits (multiple answers okay)?** | | | | | |
|  | 1: Weekly farmers market | 2: Markets | 3: Community neighbourhood stalls | 4: Street vendors in the morning or evening | 5: Moving vendors | 6: Supermarket |
|  | 7: Small shops |  |  |  |  |  |
| 4 | **Which of the food shopping possibilities do you consider having an affordable range of vegetables?** | | | | | |
|  | 1: Weekly farmers market | 1: Markets | 3: Community neighbourhood stalls | 4: Street vendors in the morning or evening | 5: Moving vendors | 6: Supermarket |
|  | 7: Small shops |  |  |  |  |  |
| 5 | **On average, which of the food shopping possibilities are within a 20-minute walk from your home?** | | | | | |
|  | 1: Weekly farmers market | 2: Markets | 3: Community neighbourhood stalls | 4: Street vendors in the morning or evening | 5: Moving vendors | 6: Supermarket |
|  | 7: Small shops |  |  |  |  |  |
| 6 | **Of the food shopping possibilities within a 20-minute walk from your home, which ones have a range of fruits and vegetables?** | | | | | |
|  | 1: Weekly farmers market | 2: Markets | 3: Community neighbourhood stalls | 4: Street vendors in the morning or evening | 5: Moving vendors (hawkers) | 6: Supermarket |
| 7 | **Which of the food shopping possibilities do you consider having hygienic range of vegetables and fruits?** | | | | | |
|  | 1: Weekly farmers market | 2: Markets | 3: Community neighbourhood stalls | 4: Street vendors in the morning or evening | 5: Moving vendors (hawkers) | 6: Supermarket |
| **Work environment** | | | | | | |
| 8 | **Please circle which of the following apply to your workplace** | | | | | |
|  | 1: Nearby *(10-minute walk)* market with a range of fruits | 2: Nearby *(10-minute walk)* community neighbourhood stalls or small shop with a range of fruits | 3: Moving vendors with a range of fruits | 4: Nearby *(10-minute walk)* supermarket with a range of fruits | 5: Cafeteria with a range of fruits | 6: No nearby (*10 minutes’ walk)* place selling fruits |
| 9 | **Would you consider the cost of fruits at your workplace relatively higher or lower than the usual prices?** | | | | | |
|  | 1: Lower | | | 2: Higher | | |
| 10 | **Please circle which of the following apply to your workplace** | | | | | |
|  | 1: Nearby *(10-minute walk)* market with a range of vegetable | 2: Nearby *(10-minute walk)* community neighbourhood stalls with a range of vegetables | 3: Moving vendors with a range of vegetables | 4: Nearby *(10-minute walk)* supermarket with a range of vegetables | 5: Cafeteria with a range of vegetables | 6: No nearby (*10 minutes’ walk)* place selling vegetables |
| 11 | **Would you consider the cost of vegetables at your workplace relatively higher or lower than the usual prices?** | | | | | |
|  | 1: Lower | | | 2: Higher | | |
| 12 | **Does your workplace cafeteria or restaurant serve at least 1 portion of vegetables on meals** | | | | | |
|  | 1: Yes | | | 2: No | | |
| **Social environment** | | | | | | |
| 13 | **Please circle on the individuals within your social network who influence your food choices (offer dietary advice, or you choose foods because of them)** | | | | | |
|  | 1: Family - children | 2: Family - husband | 3: Family - mothers | 4: Relatives | 5: Friends | 6: Workmates (colleagues) |
|  | 7: Community groups – e.g. church groups |  |  |  |  |  |
| **Virtual environment** | | | | | | |
| 14 | **Please circle on which information sources have an influence your dietary choices** | | | | | |
|  | 1: Social media – WhatsApp groups, Facebook | 2: Celebrities | 3: Mainstream media – Tv and radio health talk shows | 4: Self-proclaimed street nutrition experts | None |  |
| 15 | How often do you come across nutrition information | | | | | |
|  | 1: Never | 2: Rarely | 3: Sometimes | 4: Always |  |  |
| 16 | **On average, in a month, how often do you eat out of home (in a restaurant or takeaway)** | | | | | |
|  | 1: Never | 2: Rarely | 3: Sometimes | 4: Always |  |  |
