# Supplementary material for: The conceptual framework for a combined food literacy and physical activity intervention to optimize metabolic health among women of reproductive age in urban Uganda
Source: BMC Public Health. 2022 Feb 18;22:351. doi: 10.1186/s12889-022-12740-w (PMC8856934; doi:10.1186/s12889-022-12740-w)
Supplement: Supplementary file 8 — Additional file 8. [file 12889_2022_12740_MOESM8_ESM.docx]

**Additional file 8:** self-monitoring tool to track PA, fruit, and vegetable intake

**Guide to setup a SMART physical activity goals**

| **Questionnaire guide to setup a SMART goal**  **General tips**  **Refer to PA triangle, consider available opportunities in your environment, consider your routine, for more tips – see practical tips to make physical activity a part of your daily life** | | | | | | | |
| --- | --- | --- | --- | --- | --- | --- | --- |
|  | **Day 1 (Example of a PA plan)** | **Day 2** | **Day 3** | **Day 4** | **Day 5** | **Day 6** | **Day 7** |
| Create if then PA plan | If I jump the rope for 10 minutes without a pause every day, it will contribute to reduction of my high blood pressure back to normal |  |  |  |  |  |  |
| S – specific | Walk for 30 minutes without a pause, twice a week |  |  |  |  |  |  |
| M - measurable | 30 minutes (time using a watch or phone) |  |  |  |  |  |  |
| A – Attainable | I know that I have enough time in my week schedule to be able to reach this goal |  |  |  |  |  |  |
| R - Realistic | I am not very physically active at this moment, so walking for 30 minutes is not a too easy goal, but it’s still realistic. |  |  |  |  |  |  |
| T – Time bound | I want to reach this goal within one months |  |  |  |  |  |  |
| State when exactly you will accomplish your planned PA | on Saturday evening |  |  |  |  |  |  |
| State exactly where you will perform your PA | when coming back from market |  |  |  |  |  |  |
| State with whom you will do your planned PA | With my children |  |  |  |  |  |  |
| Participants write down at least two thing in their daily lives that could be a barrier to reaching their PA goal |  |  |  |  |  |  |  |
| Write down 1 or more concrete solutions for the barriers you wrote down |  |  |  |  |  |  |  |
| **Monitoring of goals** | | | | | | | |
| Write down whether you achieved your goal (those with smart phones – record number of steps from app/minutes) |  |  |  |  |  |  |  |
| Note down one factor that enabled you to achieve your goal |  |  |  |  |  |  |  |
| Note down one difficult situation- challenge you faced that deterred you from achieving your set goal |  |  |  |  |  |  |  |
| Provide feedback on how partners (men) take on the women involvement in the programme & propositions from the programme |  |  |  |  |  |  |  |

**Guide to setup a SMART fruit intake daily goal**

| **Questionnaire guide to setup a SMART goal**  **General tips**   - Refer to healthy food plate - Keep track of the meal opportunities and limitations, your routine/environment exposes you to. Plan accordingly - For more tips – see visual “make vegetables and fruits part of your day”. | | | **Days across the week** | | | | | | |
| --- | --- | --- | --- | --- | --- | --- | --- | --- | --- |
| **Here is an example of a fruit intake plan** | | | **Day 1** | **Day 2** | **Day 3** | **Day 4** | **Day 5** | **Day 6** | **Day 7** |
| Create if then PA plan | If I eat one portion of a fruit a day, it will contribute to reduction of my high blood pressure back to normal | |  |  |  |  |  |  |  |
| S – specific | Eat at least one portion of fruit daily | Eat at least one medium sized banana |  |  |  |  |  |  |  |
| M - measurable | One portion | One medium banana |  |  |  |  |  |  |  |
| A – Attainable | Fruits are expensive but I will plan to shop from affordable sources e.g.; weekly farmers markets | I will buy fruits from weekly farmers markets, and I will be packing a fruit when going to work |  |  |  |  |  |  |  |
| R - Realistic | I have not been eating fruits daily but one portion a day is possible | |  |  |  |  |  |  |  |
| T – Time bound | I want to reach this goal within one month | |  |  |  |  |  |  |  |
| State when exactly you will accomplish your goal | Eat a fruit as a mid-morning snack | I will eat a banana between 10am - 11am |  |  |  |  |  |  |  |
| State exactly where you will perform your fruit intake goal | When at work | |  |  |  |  |  |  |  |
| State with whom you will take your fruit | With my workmates | |  |  |  |  |  |  |  |
| Participants write down at least one thing in their daily lives that could be a barrier to reaching their fruit intake goal |  |  |  |  |  |  |  |  |  |
| Write down 1 or more concrete solutions for the barriers you wrote down |  |  |  |  |  |  |  |  |  |
| **Monitoring of goals** | | | | | | | | | |
| Write down whether you achieved your goal |  |  |  |  |  |  |  |  |  |
| Note down one factor that enabled you to achieve your goal or that deterred you from achieving it |  |  |  |  |  |  |  |  |  |
| Note down one difficult situation- challenges you faced |  |  |  |  |  |  |  |  |  |

**Guide to setup a SMART vegetable intake daily goal**

| **Questionnaire guide to setup a SMART goal**  **General tips**   - Refer to healthy food plate - Keep track of the meal opportunities and limitations, your routine/environment exposes you to. Plan accordingly - For more tips – see visual “make vegetables and fruits part of your day”. | | | **Days across the week** | | | | | | |
| --- | --- | --- | --- | --- | --- | --- | --- | --- | --- |
| **Here is an example of a vegetable intake plan** | | | **Day 1** | **Day 2** | **Day 3** | **Day 4** | **Day 5** | **Day 6** | **Day 7** |
| Create if then PA plan | If I eat one portion of a vegetable a day, it will contribute to reduction of my high blood pressure back to normal | |  |  |  |  |  |  |  |
| S – specific | Eat at least one portion of vegetable daily | Eat at least two medium sized carrots |  |  |  |  |  |  |  |
| M - measurable | One portion | Two medium sized carrots |  |  |  |  |  |  |  |
| A – Attainable | Carrots are expensive but I will plan to shop from affordable sources e.g.; markets | I will buy carrots from markets, and I will be packing carrots when going to work |  |  |  |  |  |  |  |
| R - Realistic | I have not been eating vegetables daily but one portion a day is possible | |  |  |  |  |  |  |  |
| T – Time bound | I want to reach this goal within one month | |  |  |  |  |  |  |  |
| State when exactly you will accomplish your goal | Eat carrots as a mid-morning snack | |  |  |  |  |  |  |  |
| State exactly where you will perform your vegetable intake goal | When at work | |  |  |  |  |  |  |  |
| State with whom you will take your vegetable | With my workmates | |  |  |  |  |  |  |  |
| Participants write down at least one thing in their daily lives that could be a barrier to reaching their vegetable intake goal |  | |  |  |  |  |  |  |  |
| Write down 1 or more concrete solutions for the barriers you wrote down |  | |  |  |  |  |  |  |  |
| **Monitoring of the set goals** | | | | | | | | | |
| Write down whether you achieved your goal |  | |  |  |  |  |  |  |  |
| Note down one factor that enabled you to achieve your goal or that deterred you from achieving it |  | |  |  |  |  |  |  |  |
| Note down one difficult situation- challenges you faced |  | |  |  |  |  |  |  |  |
