# Supplementary material for: The conceptual framework for a combined food literacy and physical activity intervention to optimize metabolic health among women of reproductive age in urban Uganda
Source: BMC Public Health. 2022 Feb 18;22:351. doi: 10.1186/s12889-022-12740-w (PMC8856934; doi:10.1186/s12889-022-12740-w)
Supplement: Supplementary file 9 — Additional file 9. [file 12889_2022_12740_MOESM9_ESM.docx]

**Additional file 9: Fruit and vegetable screener**

**Date** |__|__|/|__|__|/202_

**Questions on consumption of fruits and vegetables**

These questions explore intake of fruits and vegetables.

This is not a test! There are no wrong answers. Just think about how you usually do things.

**How often have you done the following actions in the last month?** Please tick or circle in the box that provides the best answer for each question.

**1: Please indicate how often you consume at least one portion of fruits**

| 1 portion of fruit is equal to 1 banana (bogoya or 2 ndinzi), orange, mango or a slice of watermelon or pineapple | 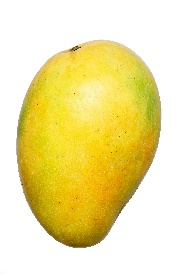[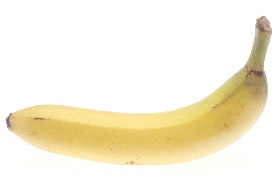](https://www.google.com/url?sa=i&url=https%3A%2F%2Fcommons.wikimedia.org%2Fwiki%2FFile%3ABanana_(1).jpg&psig=AOvVaw2bOuWa-e-PPx2PaLCTgvh6&ust=1590151697647000&source=images&cd=vfe&ved=0CAIQjRxqFwoTCIjgtfL-xOkCFQAAAAAdAAAAABAO)  What is a portion – 1 banana (bogoya), mango |
| --- | --- |

| 1: Once in a month | 2: one time a week | 3: two times a week | 4: three times a week | 5: four times a week | 6: five times a week | 7: six times a week | 8: one time a day | 9: two or more times a day |
| --- | --- | --- | --- | --- | --- | --- | --- | --- |

**2: Please indicate how often you consume at least one portion of leafy vegetables**

| Four heaped tablespoons (one handful) of cooked green leafy veggies like cabbage, sukuma, nakati, dodo, spinach, boo, amalakwang, otigo, jobiyo, | 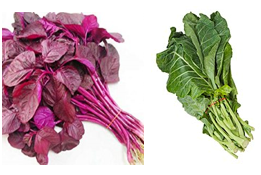  What is a portion – 4 heaped table spoons or 1 handful |
| --- | --- |

| 1: Once in a month | 2: one time a week | 3: two times a week | 4: three times a week | 5: four times a week | 6: five times a week | 7: six times a week | 8: one time a day | 9: two or more times a day |
| --- | --- | --- | --- | --- | --- | --- | --- | --- |

**3: Please indicate how often you consume at least one portion of vegetables other than leafy vegetables**

| Three heaped tablespoons (one handful) of veggies like sliced carrots, cucumber, green pepper, eggplant, mixed vegetables | 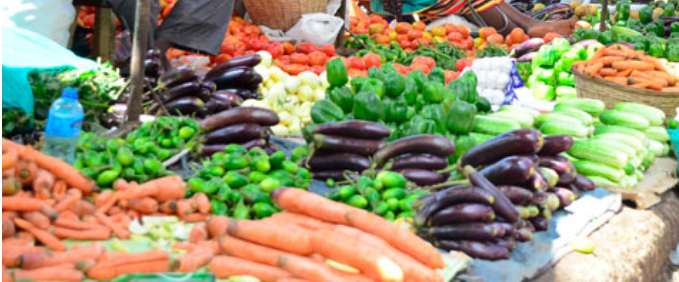  What is a portion – 3 heaped table spoons or 1 handful |
| --- | --- |

| 1: Once in a month | 2: one time a week | 3: two times a week | 4: three times a week | 5: four times a week | 6: five times a week | 7: six times a week | 8: one time a day | 9: two or more times a day |
| --- | --- | --- | --- | --- | --- | --- | --- | --- |
