# Supplementary material for: The conceptual framework for a combined food literacy and physical activity intervention to optimize metabolic health among women of reproductive age in urban Uganda
Source: BMC Public Health. 2022 Feb 18;22:351. doi: 10.1186/s12889-022-12740-w (PMC8856934; doi:10.1186/s12889-022-12740-w)
Supplement: Supplementary file 10 — Additional file 10. [file 12889_2022_12740_MOESM10_ESM.docx]

**Additional file 10: Food literacy questionnaire**

**Date** |__|__|/|__|__|/202_

**Socio demographic data**

| **1** | Name | | **…………………………………………………..** | |
| --- | --- | --- | --- | --- |
| **2** | Age | | **……………………………………………………** | |
| **3** | What is the highest level of education you have attained | | | |
|  | 1: None | 2: Primary seven | 3: 0 level certificate | 4: A Level certificate |
|  | 5: Higher institution -certificate | 6: Higher institution - diploma | 7: University - bachelor’s degree | 8: University -master’s degree |
|  | 9: University- PhD degree |  |  |  |
| **6** | Employment status | | | |
|  | 1: Iam a student | 2: Not employed | 3 Employed |  |
| **7** | What is your occupation |  | | |

**Food literacy questions**

These questions explore how you plan and prepare food for you and/or your family.

This is not a test! There are no wrong answers. Just think about how you usually do things.

**How often have you done the following actions in the last month?** Please tick or circle in the box that provides the best answer for each question.

**Questions about planning and management**

What is your opinion on the following?

| **Assessment question** | | **Likert scale** | | | |
| --- | --- | --- | --- | --- | --- |
| 1 | What is your opinion on this statement?  ‘What I eat has an impact on my health and wellbeing’ | 1: Strongly Disagree | 2: Somewhat disagree | 3: Somewhat agree | 4: Strongly Agree |
| 2 | How often do you think about health when deciding on what to eat | 1: Never | 2: Rarely | 3: Sometimes | 4: Always |

| 3 | Which of following food combinations do you consider as a typical balanced meal | 1:  Matooke,  Rice,  Chicken,  One banana | 2:  Matooke, Rice,  Beans,  One banana | 3:  Matooke,  Rice,  Greens (bugga),  One banana | 4:  Matooke,  Beans or chicken,  Greens (bugga),  One banana | 5:  Not sure |
| --- | --- | --- | --- | --- | --- | --- |
| 4 | How often do you plan meals ahead of time? | 1: Never | 2: Rarely | | 3: Sometimes | 4: Always |
| 5 | Do you normally have a weekly menu? | 1: Never | 2: Rarely | | 3: Sometimes | 4: Always |
| 6 | How often do you plan the meals to include all food groups? | 1: Never | 2: Rarely | | 3: Sometimes | 4: Always |
| 7 | How easy is it for you to plan a meal including at-least one food item from each of the food groups on a limited budget | 1: Very difficult | 2: Somehow difficult | | 3: Somehow easy | 4: Always easy |
| 8 | How often do you think about vegetables when deciding what to eat? | 1: Never | 2: Rarely | | 3: Sometimes | 4: Always |
| 9 | How often do you think about fruits when deciding what to eat? | 1: Never | 2: Rarely | | 3: Sometimes | 4: Always |

**Questions about selection**

| 10 | How often do you make a food shopping list before going to shop? | | 1: Never | 2: Rarely | 3: Sometimes | 4: Always | |
| --- | --- | --- | --- | --- | --- | --- | --- |
| 11 | How often do you purchase vegetables even if you have limited money? | | 1: Never | 2: Rarely | 3: Sometimes | 4: Always | |
| 12 | How often do you purchase fruits, even if you have limited money? | | 1: Never | 2: Rarely | 3: Sometimes | 4: Always | |
| **13** | **On average how long do you stock up on the following food items** | | | | | | |
| 13a | Leafy vegetables | 1: Do not stock (buy for immediate consumption) | 2: Daily | 3: two days | 4: three days | 5: four to five days | 6: weekly |
| 13b | Other vegetables (carrots, cucumbers, ovacado, tomatoes, eggplants etc) | 1: Do not stock (buy for immediate consumption) | 2: Daily | 3: two days | 4: three days | 5: four – five days | 6: weekly |
| 13c | Fruits | 1: Do not stock (buy for immediate consumption) | 2: Daily | 3: two days | 4: three days | 5: four– five days | 6: weekly |

| **14** | **How do you normally store your fruits and vegetables (multiple options possible)** | | | | |
| --- | --- | --- | --- | --- | --- |
|  | 1: In a fridge | 2: On a floor in the house | 3: In a basket | 4: on the shelves | 5: Others specify  …………………………………………… |

**Questions about preparation**

| 15 | How often do you cook/eat meals prepared at home? | 1: Never | 2: Rarely | 3: Sometimes | 4: Always |
| --- | --- | --- | --- | --- | --- |
| 16 | How often do you prepare vegetables at each main meal (e.g., pasted vegetables? a side dish) | 1: Never | 2: Rarely | 3: Sometimes | 4: Always |
| 17 | What is your opinion on this statement?  ‘Vegetable preparation is too much work’ | 1: Strongly agree | 2: Somehow agree | 3.Somehow disagree | 4: Strongly disagree |
| 18 | How confident do you feel about cooking a variety of healthy and tasty vegetables? | 1: Not confident | 2: Low | 3: Moderate | 4: High |
| 19 | How would you rate your ability to cook vegetables in at least three different ways? e.g. steaming, stir frying, pasting or in different dishes | 1: Not confident | 2: Low | 3: Moderate | 4: High |
| 20 | How often do you try out new vegetable recipes? | 1: Never | 2: Rarely | 3: Sometimes | 4: Always |
| 21 | Rate your confidence in changing recipes (written or unwritten) to make them healthier | 1: Not confident | 2: Low | 3: Sometimes | 4: Always |
| 22 | How confident are you that vegetables prepared at home don’t contain pesticide residues | 1: Not confident | 2: Low | 3: Moderate | 4: High |
| 23 | How confident are you that fruits prepared at home don’t contain pesticide residues | 1: Not confident | 2: Low | 3: Moderate | 4: High |

**Questions about eating behavior**

| 24 | How many portions of vegetables is ministry of health and world health organisation is recommending people to eat? (a portion = 3 heaped table spoons or 1 handful) | 1: two or more portions a day | 2: one portion a day | 3: five portions a week | 4: three portions a week | 5: one portion a week | 6: Not sure |
| --- | --- | --- | --- | --- | --- | --- | --- |
| 25 | How many portions of fruits ministry of health and world health organisation is recommending people to eat? (1 portion of fruit is equal to 1 medium piece of banana, orange, mango or a slice of watermelon or pineapple) | 1: two or more portions a day | 2: one portion a day | 3: five portions a week | 4: three portions a week | 5: one portion a week | 6: Not sure |

**26: Please indicate how often you consume at least one portion of fruits**

| 1 portion of fruit is equal to 1 banana (bogoya or 2 ndinzi), orange, mango or a slice of watermelon or pineapple | 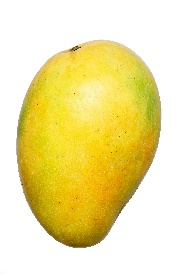[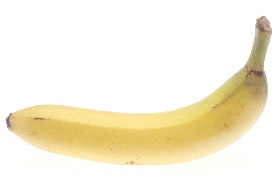](https://www.google.com/url?sa=i&url=https://commons.wikimedia.org/wiki/File:Banana_(1).jpg&psig=AOvVaw2bOuWa-e-PPx2PaLCTgvh6&ust=1590151697647000&source=images&cd=vfe&ved=0CAIQjRxqFwoTCIjgtfL-xOkCFQAAAAAdAAAAABAO)  What is a portion – 1 banana (bogoya), mango |
| --- | --- |

| 1: Once in a month | 2: one time a week | 3: two times a week | 4: three times a week | 5: four times a week | 6: five times a week | 7: six times a week | 8: one time a day | 9: two or more times a day |
| --- | --- | --- | --- | --- | --- | --- | --- | --- |

**27: Please indicate how often you consume at least one portion of leafy vegetables**

| Four heaped tablespoons (one handful) of cooked green leafy veggies like cabbage, sukuma, nakati, dodo, spinach, boo, amalakwang, otigo, jobiyo, | 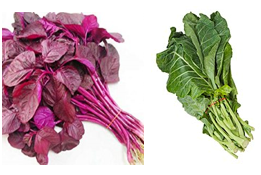  What is a portion – 4 heaped table spoons or 1 handful |
| --- | --- |

| 1: Once in a month | 2: one time a week | 3: two times a week | 4: three times a week | 5: four times a week | 6: five times a week | 7: six times a week | 8: one time a day | 9: two or more times a day |
| --- | --- | --- | --- | --- | --- | --- | --- | --- |

**28: Please indicate how often you consume at least one portion of vegetables other than leafy vegetables**

| Three heaped tablespoons (one handful) of veggies like sliced carrots, cucumber, green pepper, eggplant, mixed vegetables | 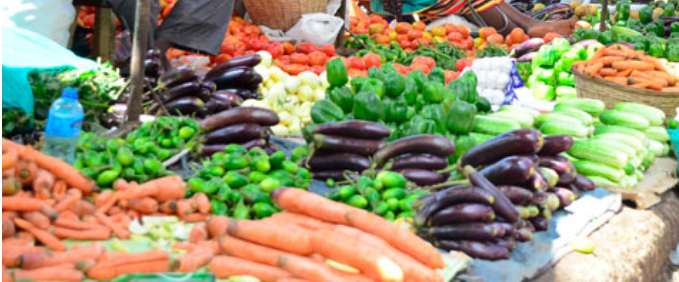  What is a portion – 3 heaped table spoons or 1 handful |
| --- | --- |

| 1: Once in a month | 2: one time a week | 3: two times a week | 4: three times a week | 5: four times a week | 6: five times a week | 7: six times a week | 8: one time a day | 9: two or more times a day |
| --- | --- | --- | --- | --- | --- | --- | --- | --- |

| **29** | **How easy is it for you to eat fruits and vegetables when you find yourself in the following situations** | | | | |
| --- | --- | --- | --- | --- | --- |
| 29a | When at work | 1: Very difficult | 2: Somewhat difficult | 3: Somewhat easy | 4: Always easy |
| 29b | When traveling | 1: Very difficult | 2: Somewhat difficult | 3: Somewhat easy | 4: Always easy |
| 29c | When you are busy | 1: Very difficult | 2: Somewhat difficult | 3: Somewhat easy | 4: Always easy |
| 30 | How often do you pack fruits when going to work? | 1: Never | 2: Rarely | 3: Sometimes | 4: Always |
| 31 | How often do you pack vegetables when going to work? | 1: Never | 2: Rarely | 3: Sometimes | 4: Always |
| 32 | How often do you eat vegetables as snacks (between meals)? e.g. avocado, carrots, tomatoes, cucumbers | 1: Never | 2: Rarely | 3: Sometimes | 4: Always |
| 33 | How often do you eat fruits as a snack (in between meals)? | 1: Never | 2: Rarely | 3: Sometimes | 4: Always |

**Questions on nutrition information**

| 34 | When you have questions on what food to eat for better health, do you know where to ﬁnd this information? | 1: Never | 2: Rarely | 3: Sometimes | 4: Always |
| --- | --- | --- | --- | --- | --- |
| 35 | There is a lot of information available on healthy eating today. How well do you manage to choose the information relevant to you? | 1: Very difficult | 2: Somewhat difficult | 3: Somewhat easy | 4: Always easy |
| 36 | How easy is it for you to judge if healthy eating information shared on various platforms can be trusted? | 1: Very difficult | 2: Somewhat difficult | 3: Somewhat easy | 4: Always easy |
